# Variant-Specific Temporal Transcriptomic Responses of Ex Vivo Dog Lung Explants to SARS-CoV-2 Delta and Omicron Infection

**DOI:** 10.3390/ijms27209319

**Published:** 2026-10-20

**Authors:** Anuradha Panwar, Ashwin Ashok Raut, Amod Kumar, Sonal Sharma, Abhishek Jangir, Suneela Saran, Pushpendra Kumar, Piyush Joshi, Raj Kumar Singh, Anamika Mishra

**Affiliations:** 1 ICAR- National Institute of High Security Animal Diseases, Bhopal 462022, India; anuradhapanwar888@gmail.com (A.P.); ashwin.nihsad@gmail.com (A.A.R.); sonalsharmaa2017@gmail.com (S.S.); absedhwal@gmail.com (A.J.); piyushbiotech2227@gmail.com (P.J.); 2 ICAR- National Bureau of Animal Genetic Resources, Karnal 132001, India; amodvet@gmail.com; 3 AASRA, Bhopal 462008, India; suneela.saran6@gmail.com; 4 Department of Animal Genetics and Breeding, ICAR-Indian Veterinary Research Institute, Izatnagar, Bareilly 243122, India; pushpendra64@gmail.com (P.K.); raj.singh@fao.org (R.K.S.)

**Keywords:** Delta variant, dog lung explants, host–pathogen interactions, Omicron variant, SARS-CoV-2, transcriptomics

## Abstract

The rapid evolution and emergence of SARS-CoV-2 variants have posed significant public health challenges due to enhanced transmissibility and diverse clinical outcomes. While the effects of these variants in humans have been extensively investigated, their impact on domestic animals remains insufficiently understood. Given the close interaction between humans and dogs, along with documented cases of human-to-animal transmission and the potential role of companion animals as reservoirs for viral evolution, understanding the molecular dynamics of SARS-CoV-2 infection in dogs is of considerable importance. However, in vivo infection studies in non-laboratory animal species are highly resource intensive, time-consuming, require ABSL3 facilities, and involve ethical constraints, limiting their widespread application. In this context, ex vivo culture systems provide a practical and physiologically relevant alternative. Therefore, in the present study, we employed an ex vivo lung explant culture model derived from domestic dogs to investigate host responses to the SARS-CoV-2 Delta and Omicron variants. Comprehensive transcriptomic profiling across multiple time points post-infection revealed significant alterations in genes associated with cell adhesion, structural components, extracellular matrix (ECM) organization and regulation, immune responses, mitochondrial activity, oxidative phosphorylation, and ribosomal function. Both variants induced marked downregulation of ECM-associated and T-cell-related genes, suggesting disruption of tissue integrity and suppression of adaptive immune responses. The Delta variant was characterized by early and sustained suppression of immune-associated signaling pathways, particularly T-cell receptor signaling, whereas the Omicron variant induced stronger activation of genes associated with mitochondrial activity, oxidative phosphorylation, and translational responses. At later stages of infection, especially at 24 h post-infection (hpi), canine lung explants exhibited increased expression of genes related to tissue repair, epithelial maintenance, mitochondrial function, and ribosomal activity, suggesting activation of compensatory responses aimed at maintaining cellular stability under viral stress. Notably, several hub genes were identified, including *CD2*, *CD3D*, *CD3E*, *CD27*, *PTPRC*, *ZAP70*, *SYK*, and *LCP2* in Delta infection, and *FLT3LG*, *MRPL2*, *MRPL4*, *MRPS11*, *MRPS12*, *RPS13*, and *RPS5* in Omicron infection, which are involved in critical pathways associated with immune signaling, mitochondrial function, metabolic regulation, and protein synthesis. Furthermore, the observed mitochondrial and ribosomal responses may indicate that therapeutic strategies aimed at supporting mitochondrial function and modulating excessive inflammation could potentially help reduce lung damage during severe infection. Overall, this study provides important insights into the transcriptomic response of dog lung explants following exposure to SARS-CoV-2 Delta and Omicron variants and also highlights the utility of ex vivo canine lung explant cultures as a valuable platform for studying host–pathogen interactions.

## 1. Introduction

Since the emergence of severe acute respiratory syndrome coronavirus 2 (SARS-CoV-2) in Wuhan, China, and its subsequent classification as a public health emergency of international concern in January 2020 [1] and a global pandemic in March 2020 by the World Health Organization (WHO), significant efforts have been directed toward understanding its transmission dynamics, host range, and evolutionary potential [2]. In this context, animals have been extensively investigated for their possible involvement in viral maintenance and spread. Companion animals, particularly domestic cats and dogs, have attracted considerable attention due to their close and prolonged contact with humans [3]. Experimental infection studies have confirmed that both cats and dogs are susceptible to SARS-CoV-2 [4,5,6,7], and numerous field investigations have documented natural infections in these species across different geographical regions [2,8,9,10,11,12,13,14,15,16,17,18,19,20]. Despite this susceptibility, current evidence suggests that their contribution to SARS-CoV-2 transmission remains limited [2,21]. With the exception of a suspected case involving cat-to-human transmission [22], there is no definitive evidence supporting transmission from these companion animals to humans. However, confirmed cases of animal-to-human transmission in other species, such as farmed mink and pet Syrian hamsters, demonstrate that cross-species transmission is possible under certain conditions [23,24,25,26,27,28]. Notably, outbreaks in mink farms have been associated with the emergence of viral mutations, including Y453F, highlighting the role of animal hosts in viral evolution [24,25,29]. Although some of these mutations may reduce viral fitness in human airway cells [29], their long-term implications remain uncertain, emphasizing the need for continued monitoring [30]. While the likelihood of zoonotic transmission from cats and dogs appears low, it cannot be entirely ruled out, particularly with the continuous emergence of new variants. Therefore, a detailed understanding of host responses to SARS-CoV-2 exposure in these animals is essential. High-throughput transcriptomic approaches provide valuable insights into host–pathogen interactions by enabling the identification of genes and pathways involved in immune responses and viral pathogenesis. In our previous work, we conducted a transcriptomic analysis of SARS-CoV-2 infection in cats [31], which revealed important aspects of the feline host response. Extending this line of investigation, the present study focuses on dogs to broaden our understanding of species-specific and comparative host responses among companion animals.

Commonly used in vitro systems, although useful for controlled virological studies, do not adequately replicate the complexity of intact tissues, including cell–cell interactions and extracellular matrix components. On the other hand, in vivo experiments provide comprehensive physiological insights but are often limited by ethical constraints, high costs, and the requirement for advanced biosafety facilities. Ex vivo lung explant cultures represent an intermediate approach that preserves tissue architecture and local immune characteristics while allowing experimental manipulation under controlled conditions [32]. Although these systems are limited by their short-term viability and lack of systemic immune responses, they offer a practical and biologically relevant platform for studying respiratory viral infections. In this study, we performed transcriptomic profiling of dog lung explant cultures following exposure to the SARS-CoV-2 Delta and Omicron variants at three distinct time points, enabling the investigation of temporal and variant-specific host responses under ex vivo conditions. Notably, in our previous study, a similar transcriptomic approach was applied to cats, allowing for comparative insights into species-specific responses to SARS-CoV-2 infection [31]. Differential gene expression analysis, functional enrichment, and network-based approaches were used to identify key pathways and hub genes associated with infection. Hub genes, defined by their high degree of connectivity within interaction networks, are often critical regulators of biological processes [33]. For hub genes the ROC analysis was also performed using the pROC package in R 4.4.1 [34,35]. As the analysis used the same dataset from which the hub genes were identified, the AUC values were considered exploratory measures of within-dataset discrimination rather than evidence of independent diagnostic or predictive utility and are presented as Table S4 and Figure S1 of Supplementary Materials S1. Few hub genes and additional genes identified from the transcriptomic analysis were subsequently validated by real-time PCR. Overall, this study provides insights into the transcriptomic responses of dog lung explant culture following exposure to SARS-CoV-2 variants and highlights the utility of the ex vivo lung explant model for investigating host–pathogen interactions.

## 2. Results

### 2.1. Confirmation of Viral Infection in Explant Culture

Throughout the experiment, the RPMI medium retained its original color, indicating that the explant cultures remained viable and free from contamination. Viral infection was confirmed by real-time PCR using the VIRALDTECT-II Multiplex kit. The Ct values ranged from 24 to 29 at 0 hpi and decreased to 16–22 in samples collected at 6, 12, and 24 hpi following infection with the Delta and Omicron variants. In contrast, no amplification was detected in the mock (non-infected) samples. The gradual decline in Ct values over time indicated a progressive increase in detectable viral RNA in the ex vivo dog lung explant model. The Ct values obtained at different time points for different variants were provided in Table S1 of Supplementary Materials S1.

### 2.2. RNA Seq Data Generation

FASTQ files containing raw sequencing data were obtained from mock, 6 hpi, 12 hpi, and 24 hpi samples, with data sizes ranging from 8 to 17 Gb per sample. The number of sequencing reads (in millions) and the corresponding raw data volume (Gb) for each sample are presented in Table S2 of Supplementary Materials S1.

### 2.3. Differentially Expressed Genes for Delta and Omicron Variant Infection at Different Time Points

**Figure 1:**
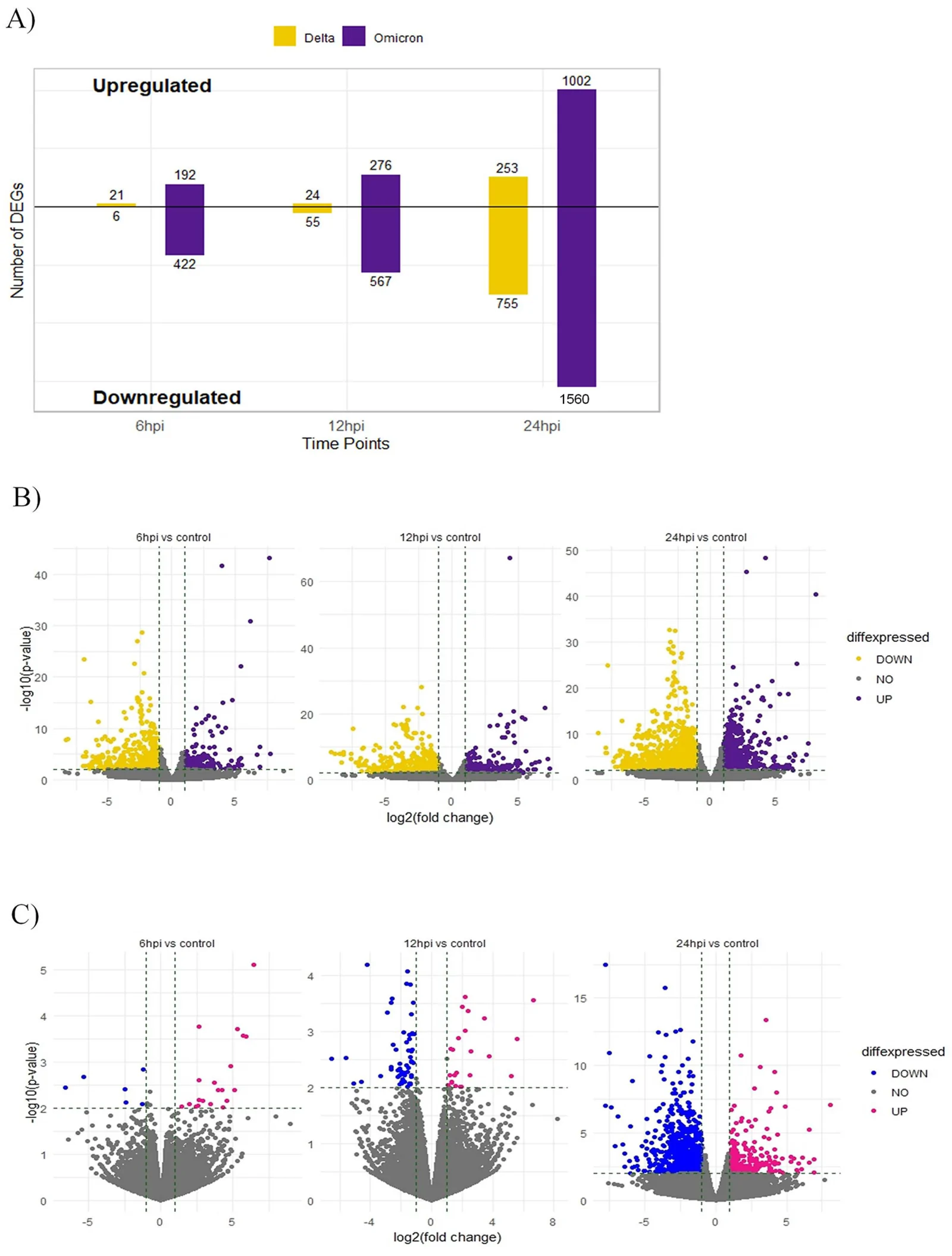
DEGs following Delta and Omicron variant infection in dog lung explant cultures. (**A**) Numbers of upregulated and downregulated DEGs at 6, 12, and 24 hpi. (**B**,**C**) Volcano plots showing DEGs following Delta and Omicron infection, respectively, compared with mock controls. DEGs were identified using a log_2_FC ≥ 1 or ≤−1 and a *p*-value ≤ 0.01.

Following SARS-CoV-2 Delta variant infection in dog lung explant culture, a total of 27, 79, and 1008 DEGs were obtained at 6, 12, and 24 hpi, respectively. Among these, 6 DEGs were downregulated and 21 DEGs were upregulated at 6 hpi, 55 DEGs were downregulated, and 24 DEGs upregulated at 12 hpi, while 755 DEGs were downregulated and 253 DEGs were upregulated at 24 hpi. Similarly, following Omicron variant infection in dog lung explant culture, 614, 843, and 2562 DEGs were identified at 6, 12, and 24 hpi, respectively. Among these, 192 DEGs were upregulated and 422 were downregulated at 6 hpi. At 12 hpi, 276 DEGs were upregulated and 567 were downregulated, while at 24 hpi, 1002 DEGs were upregulated and 1560 were downregulated (Figure 1A). Figure 1B and Figure 1C represent the volcano plot after Delta and Omicron variant infection in dog lung explant culture, respectively. The lists of unfiltered genes and filtered DEGs are provided in Supplementary Materials S3 and S4.

### 2.4. Short Time-Series Expression Miner

**Figure 2:**
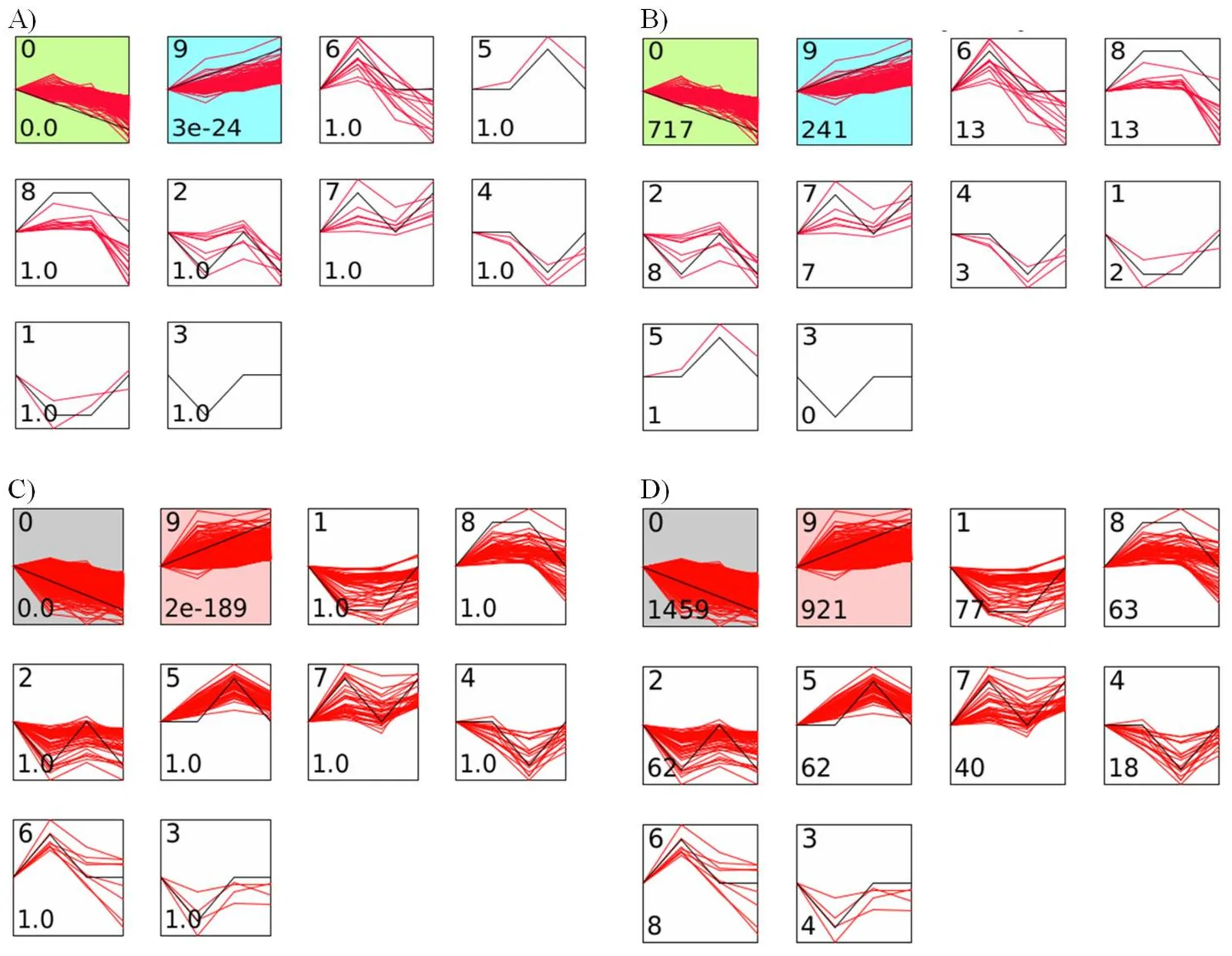
Trend analysis of gene expression in Delta- and Omicron-infected dog lung explant cultures at different time points post-infection using STEM (Short Time-series Expression Miner). (**A**) Significant temporal expression profiles (*p* ≤ 0.01) identified following Delta variant infection. (**B**) Distribution of the number of genes assigned to each expression profile after Delta variant infection. (**C**) Significant temporal expression profiles (*p* ≤ 0.01) identified following Omicron variant infection. (**D**) Distribution of the number of genes assigned to each expression profile after Omicron variant infection. Each panel represents model-derived temporal expression patterns generated by STEM. In each profile box, the number at the top-left indicates the profile ID, while the bottom-left value in (**A**,**C**) represents the *p*-value indicating statistical significance of gene enrichment. In (**B**,**D**), the bottom-left number denotes the number of genes assigned to each profile. The black line represents the mean expression trend of genes within a profile, while red lines indicate individual gene expression trajectories normalized to the model pattern. Colored backgrounds highlight statistically significant profiles as determined by STEM.

To investigate gene expression dynamics during early disease progression, the identified differentially expressed genes (DEGs) from the 6 hpi vs. Mock, 12 hpi vs. Mock, and 24 hpi vs. Mock comparisons were classified into 10 expression profiles using Short Time-series Expression Miner (STEM) v1.3.13 software. In Delta variant-infected samples, two profiles were identified as statistically significant (Figure 2A), comprising 717 genes in profile 0 and 241 genes in profile 9 (Figure 2B). Similarly, two significant expression profiles were observed following Omicron variant infection (Figure 2C), with profile 0 containing 1459 genes and profile 9 containing 921 genes (Figure 2D).

#### 2.4.1. GO and KEGG Pathway Analysis of Significant Profiles

To further elucidate the functional significance of the two significant expression profiles identified following infection with the Delta and Omicron variants, Gene Ontology (GO) and Kyoto Encyclopedia of Genes and Genomes (KEGG) pathway enrichment analyses were performed. Enrichment terms with Benjamini-adjusted *p*-values and false discovery rate (FDR) values ≤ 0.05 were considered statistically significant. For both variants, the analysis focused on Profile 0, comprising significantly downregulated differentially expressed genes (DEGs), and Profile 9, comprising significantly upregulated DEGs.

**Figure 3:**
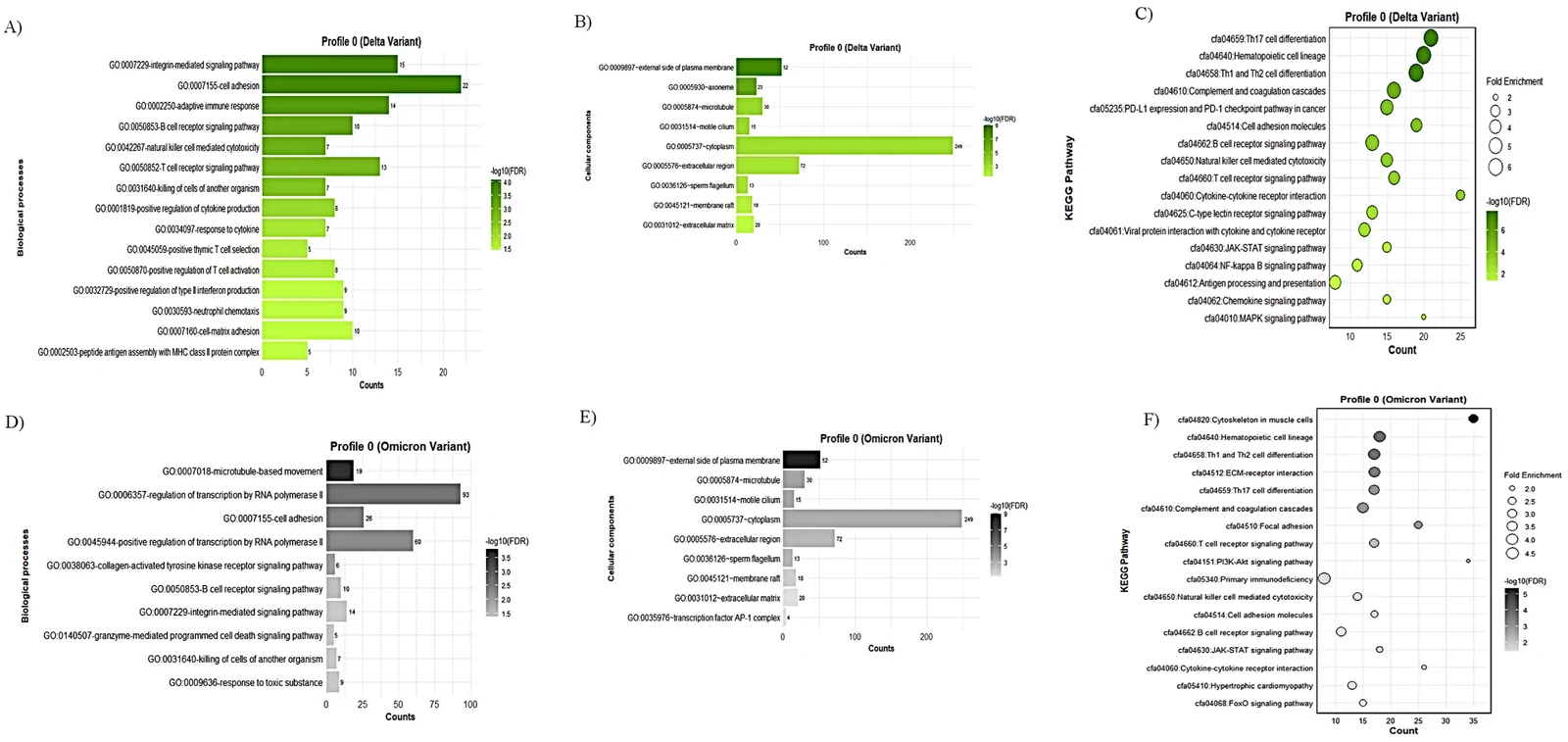
Functional enrichment of genes from Profile 0 following Delta and Omicron variant infections. (**A**–**C**) represent the Delta variant, and (**D**–**F**) represent the Omicron variant. (**A**,**D**) show enriched Biological Processes (BPs), (**B**,**E**) Cellular Components (CCs), and (**C**,**F**) KEGG pathways. Bar plots indicate gene counts, while dot plots show pathway enrichment (dot size = fold enrichment).

**Figure 4:**
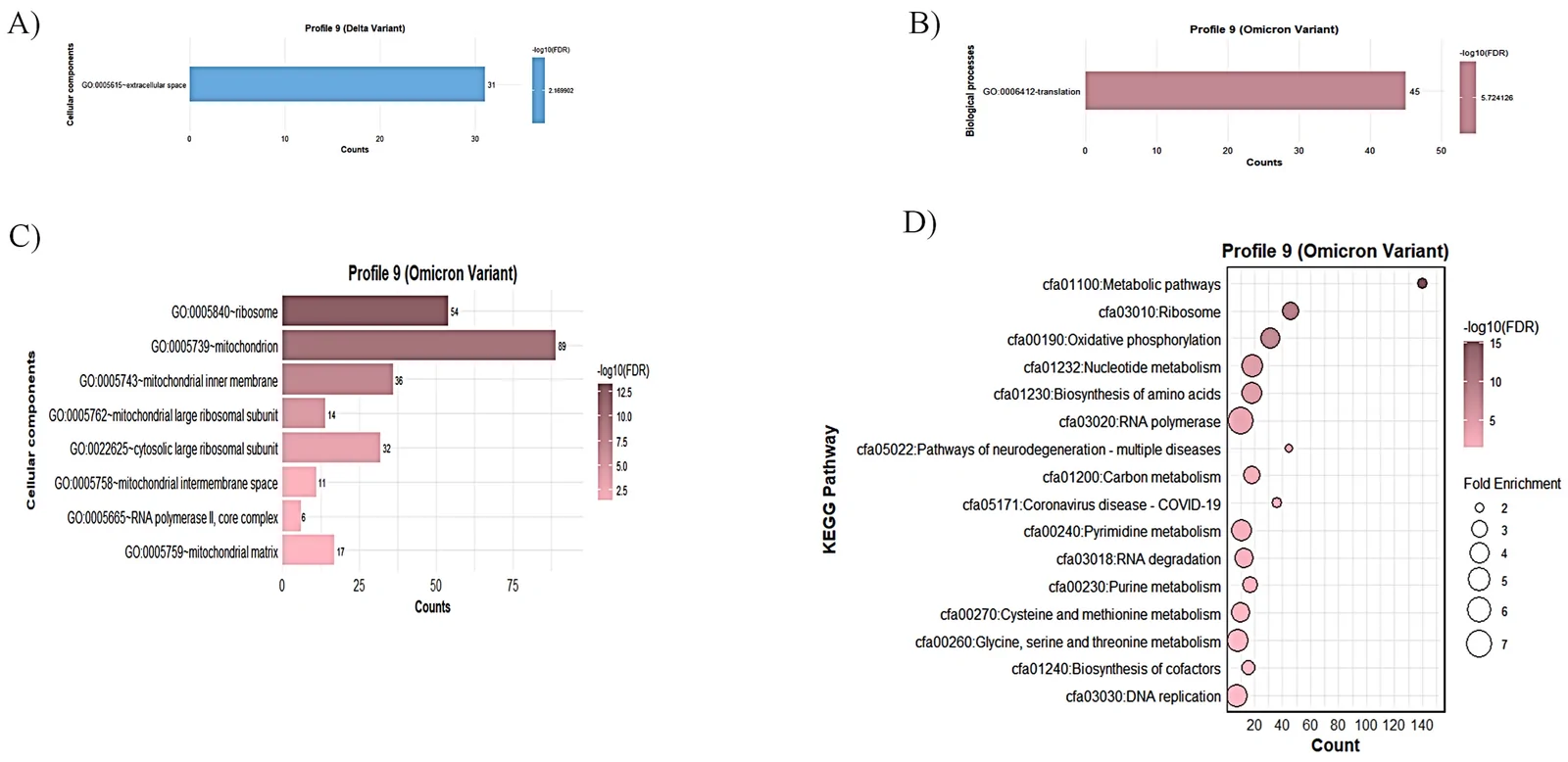
Functional enrichment of genes from Profile 9 following Delta and Omicron variant infections. (**A**) shows enriched Cellular Components (CCs) for the Delta variant. (**B**) shows enriched Biological Processes (BPs), (**C**) enriched Cellular Components (CCs), and (**D**) enriched KEGG pathways for the Omicron variant. Bar plots represent gene counts, while the KEGG dot plot shows pathway enrichment, with dot size indicating fold enrichment.

Following Delta variant infection, Profile 0 was enriched in biological processes, including adaptive immune response, B-cell receptor signaling, T-cell receptor signaling, natural killer cell-mediated cytotoxicity, and positive regulation of type II interferon production, whereas cellular components were enriched in extracellular space, matrix, and region. KEGG pathway analysis further revealed enrichment of pathways associated with cytokine–cytokine receptor interactions, JAK–STAT, chemokine, and MAPK signaling, as well as antigen processing and presentation (Figure 3A–C). In contrast, Profile 9 showed significant enrichment only for the extracellular space under cellular component analysis, with no significant biological processes or KEGG pathways identified (Figure 4A).

Following Omicron variant infection, Profile 0 was enriched in biological processes associated with cell adhesion, positive regulation of transcription by RNA polymerase II, collagen-activated tyrosine kinase receptor signaling, B-cell receptor signaling, and integrin-mediated signaling. Enriched cellular components included the external side of the plasma membrane, cytoplasm, extracellular region, and extracellular matrix. KEGG pathway analysis revealed enrichment of pathways related to Th1 and Th2 cell differentiation, Th17 cell differentiation, ECM–receptor interaction, focal adhesion, T-cell and B-cell receptor signaling, PI3K–Akt signaling, cell adhesion molecules, cytokine–cytokine receptor interactions, and JAK–STAT signaling (Figure 3D–F). In contrast, Profile 9 was enriched in the biological process of translation, and cellular components associated with ribosomes and mitochondria, including mitochondrial ribosomal subunits, the mitochondrial inner membrane, the mitochondrial matrix, and the mitochondrial intermembrane space, were enriched. The enriched KEGG pathways included metabolic pathways, ribosome, chemical carcinogenesis–reactive oxygen species, nucleotide metabolism, biosynthesis of amino acids, coronavirus disease (COVID-19), pyrimidine metabolism, and RNA degradation (Figure 4B–D).

Overall, the enrichment analysis revealed differences in the functional categories associated with the two expression profiles following infection with the Delta and Omicron variants. In both variants, Profile 0 was associated with immune-related signaling and cellular processes, although the specific enriched terms differed. In Omicron-infected explants, Profile 9 was additionally associated with translation, ribosomal components, and metabolic pathways, whereas Delta Profile 9 showed limited enrichment. Detailed GO and KEGG enrichment results for Profiles 0 and 9 following Delta and Omicron variant infections are provided in Supplementary Materials S2.

#### 2.4.2. Screening of Genes in Profile 0 Following Delta and Omicron Variant Infection in Dog Lung Explant Culture

**Figure 5:**
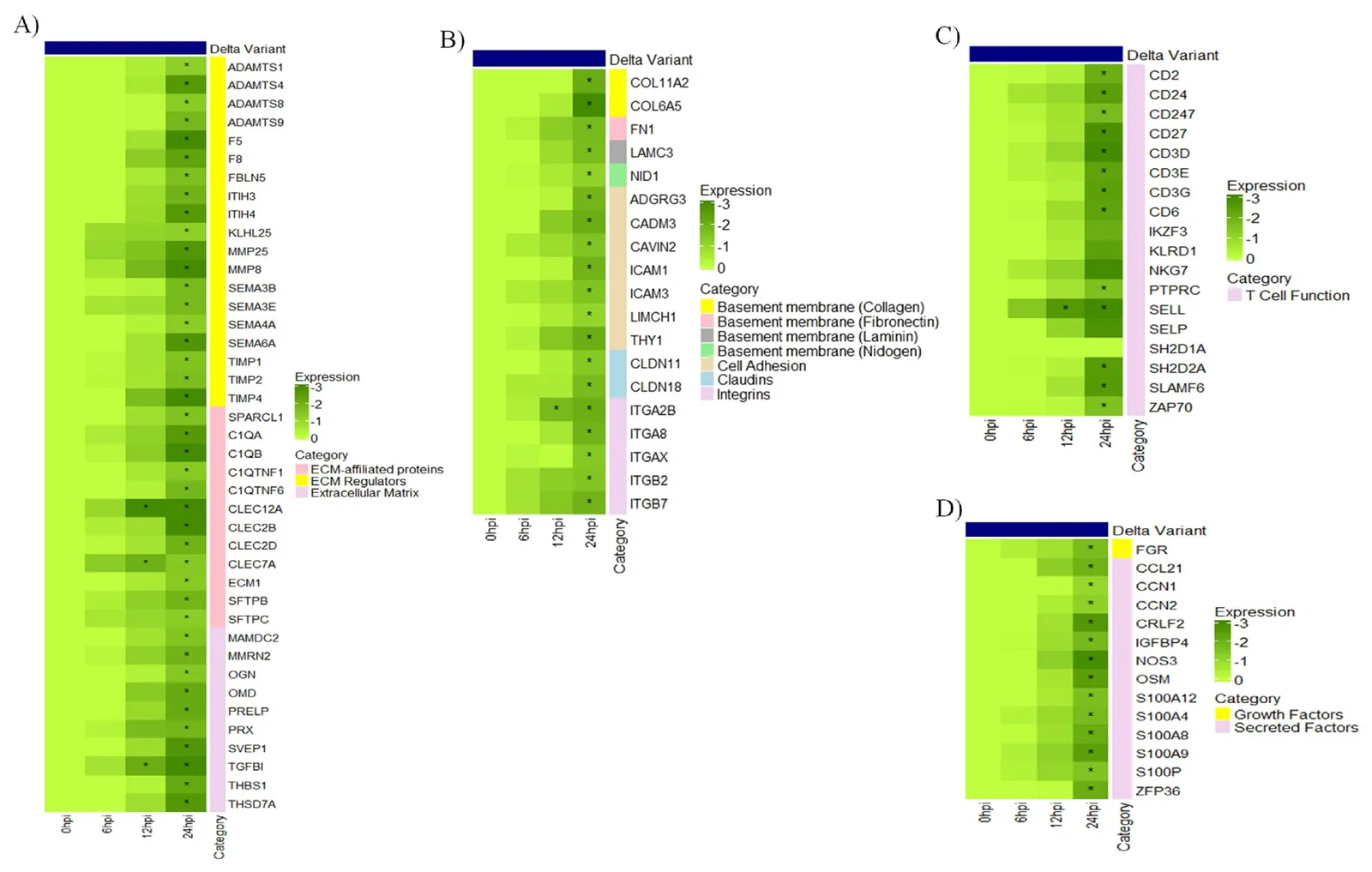
Heatmap showing the expression dynamics of genes associated with (**A**) extracellular matrix (ECM) organization and regulation, (**B**) basement membrane and cell adhesion, (**C**) T-cell function, and (**D**) growth factors and secreted signaling molecules following Delta variant infection at 0, 6, 12, and 24 hpi. These genes represent downregulated DEGs identified in Profile 0. Genes with statistically significant expression changes (*p*-value ≤ 0.01) are indicated with asterisks (*).

**Figure 6:**
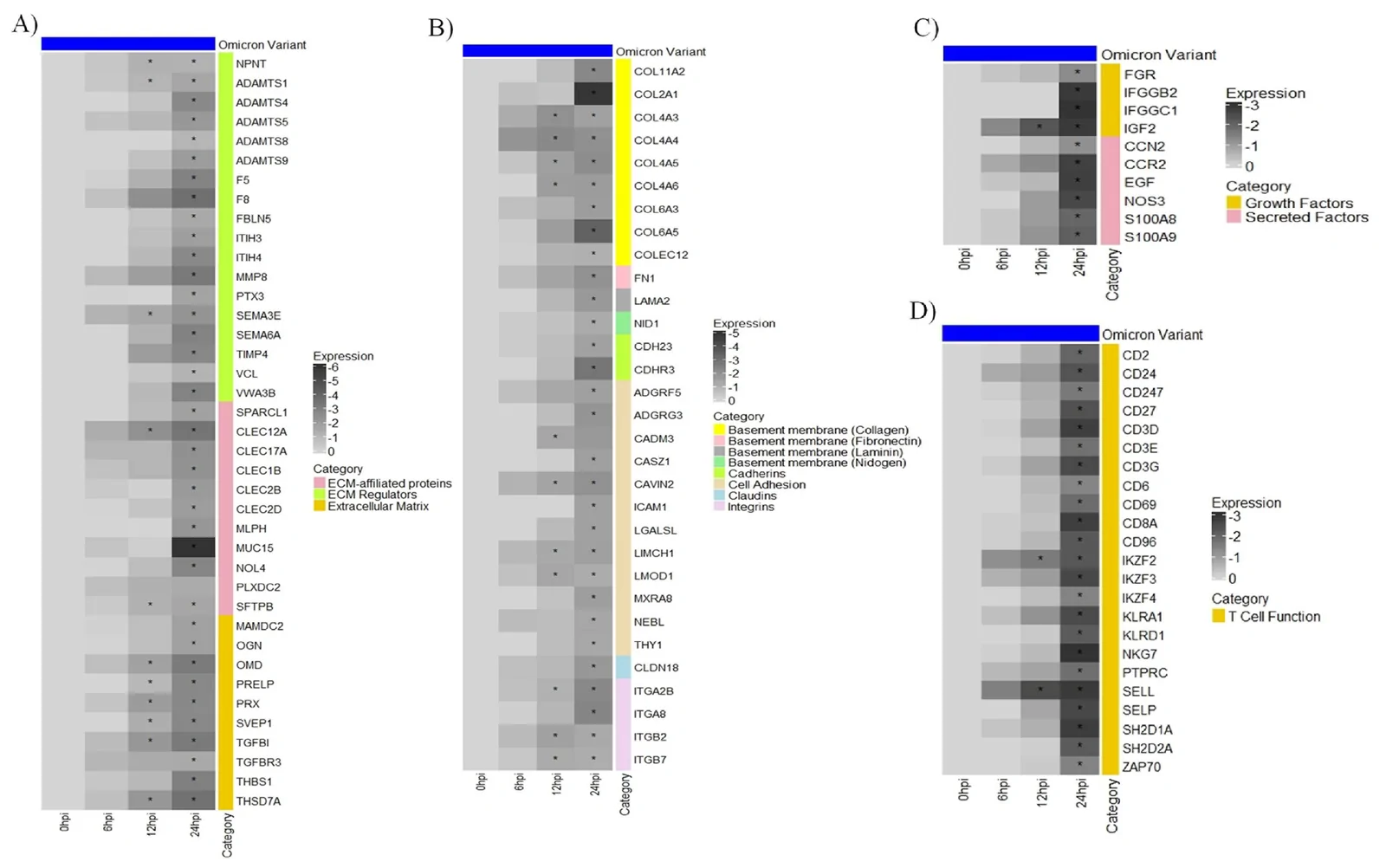
Heatmap showing the expression dynamics of genes associated with (**A**) extracellular matrix (ECM) organization and regulation, (**B**) basement membrane and cell adhesion, (**C**) growth factors and secreted factors, and (**D**) T-cell function following Omicron variant infection at 0, 6, 12, and 24 hpi. These genes represent downregulated DEGs identified in Profile 0. Genes with statistically significant expression changes (*p*-value ≤ 0.01) are indicated with asterisks (*).

Transcriptomic analysis of Profile 0 in dog lung explants infected with SARS-CoV-2 Delta and Omicron variants revealed significant downregulation of genes associated with cell adhesion, structural components, and extracellular matrix (ECM) organization. In addition, genes involved in immune response, T-cell function, growth factors, and secreted molecules were consistently suppressed in both variants. A considerable number of differentially expressed genes (DEGs) were upregulated, with a pronounced enrichment of mitochondrial and ribosomal genes observed specifically in the Omicron variant. These findings suggest that SARS-CoV-2 disrupts ECM integrity, potentially compromising lung structure and contributing to inflammatory responses [36]. Consistent with this, both variants exhibited marked downregulation of ECM and cell adhesion-related genes. Basement membrane collagen genes, including *COL2A1*, *COL11A2*, *COL4A* family members, and *COL6A5*, were significantly reduced, along with key ECM components such as *FN1*, laminins (*LAMA2*/*LAMC3*), and *NID1*. Cell adhesion molecules (*ICAM1*, *ICAM3*, *CADM3*, *THY1*) and integrins (*ITGA2B*, *ITGA8*, *ITGB7*) were also consistently downregulated, indicating disruption of cell ECM interactions and tissue integrity. Further, widespread downregulation of ECM regulatory genes, ECM-affiliated proteins, and structural components was observed in both variants. Key ECM regulators (*ADAMTS1*, *ADAMTS4*, *ADAMTS9*, *F8*), metalloproteinases (*MMP8*, *MMP25*), and associated genes (*TIMP4*, *SEMA6A*) were significantly reduced, suggesting decreased ECM remodeling activity. ECM-affiliated genes, including *CLEC* family members, *C1QA/B*, and *SPARCL1*, were also downregulated, indicating impaired ECM–immune interactions. Structural ECM components such as *OMD*, *PRELP*, *TGFBI*, *THSD7A*, and *SVEP1* showed marked reduction, reflecting compromised tissue stability. Notably, the Omicron variant exhibited stronger downregulation of several structural genes, including a pronounced decrease in *MUC15*, suggesting enhanced disruption of mucin-mediated lung defense mechanisms. In parallel, both variants induced significant suppression of immune response genes, affecting both innate and adaptive immunity. A pronounced downregulation of T-cell function genes was also observed, including key components of the T-cell receptor complex (*CD3D*, *CD3E*, *CD3G*, *CD247*) and activation markers (*CD27*, *CD2*), suggesting impaired T-cell signaling and activation. Additional genes involved in T-cell regulation and migration, such as *PTPRC*, *IKZF2/3*, and *SELL*, were also reduced, indicating disrupted T-cell differentiation and trafficking. Moreover, genes associated with growth factors and secreted molecules were significantly downregulated in both variants. Growth-related genes such as *FGR* and *IGFBP4* were reduced in the Delta variant, while a stronger suppression of *IGFBP2*, *IGF2*, and related genes was observed in the Omicron variant, suggesting impaired tissue repair capacity. Secreted factors involved in immune signaling, including *CCL21*, *CCN* family members, *NOS3*, and *EGF*, were also downregulated, indicating reduced immune cell recruitment and weakened inflammatory signaling. Overall, these findings demonstrate that both Delta and Omicron variants induce extensive suppression of ECM organization, immune function, and tissue repair pathways in dog lung explants, potentially compromising lung integrity and host defense. Figure 5 and Figure 6 represent heatmaps of differentially expressed genes across key functional categories in dog lung explant cultures infected with SARS-CoV-2 Delta and Omicron variants, including cell adhesion and structural components, extracellular matrix (ECM) organization and regulation, growth factors, secreted factors, and T cell function. The displayed genes correspond to downregulated DEGs from Profile 0. Statistically significant changes (*p* ≤ 0.01) are indicated by asterisks (*).

#### 2.4.3. Screening of Genes in Profile 9 Following Delta and Omicron Variant Infection in Dog Lung Explant Culture

**Figure 7:**
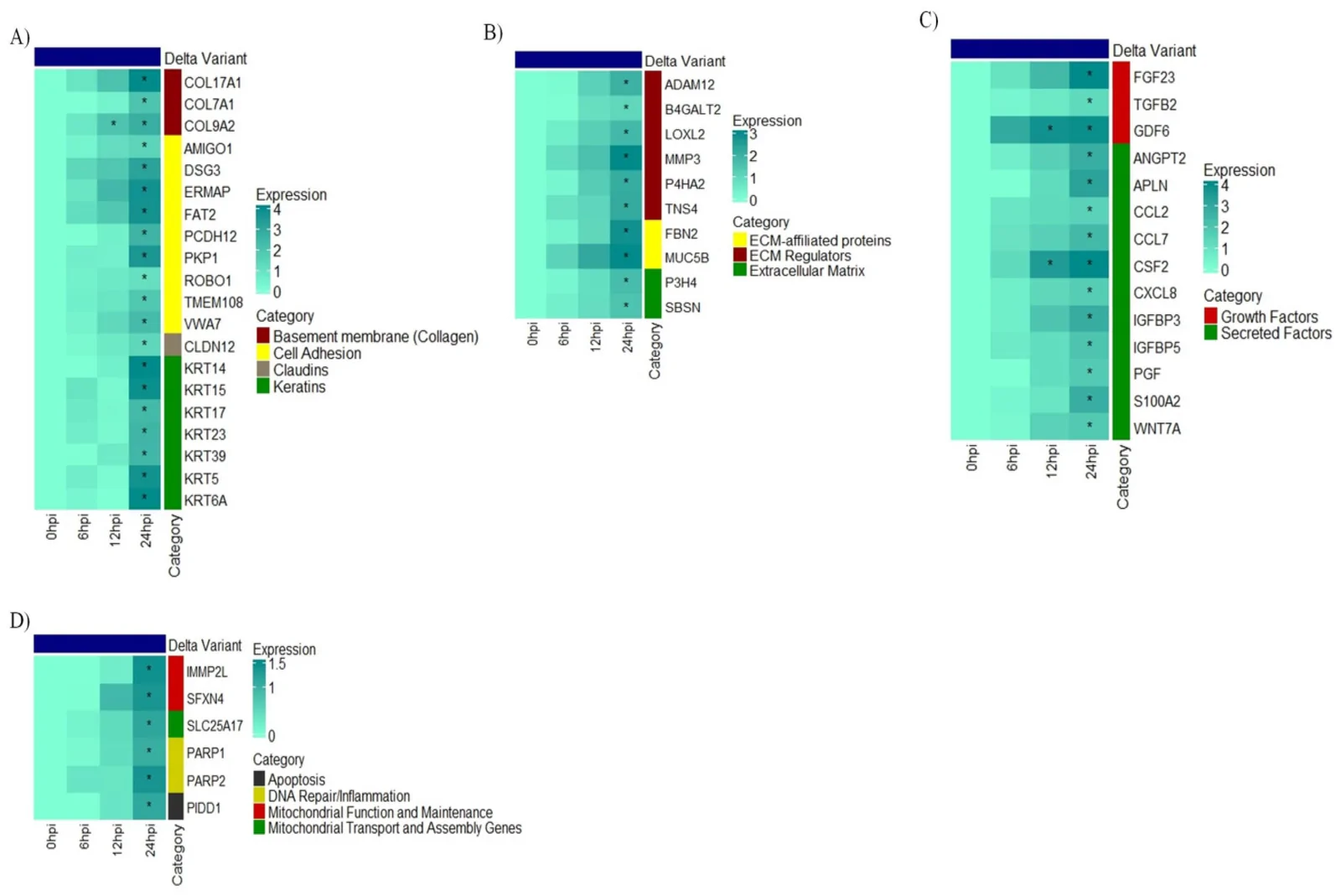
Heatmap showing the expression dynamics of genes associated with (**A**) basement membrane and cell adhesion, (**B**) extracellular matrix (ECM) organization and regulation, (**C**) growth factors and secreted factors, and (**D**) apoptosis, DNA repair/inflammation, and mitochondrial function following Delta variant infection at 0, 6, 12, and 24 hpi. These genes represent upregulated DEGs identified in Profile 9. Genes with statistically significant expression changes (*p*-value ≤ 0.01) are indicated with asterisks (*).

**Figure 8:**
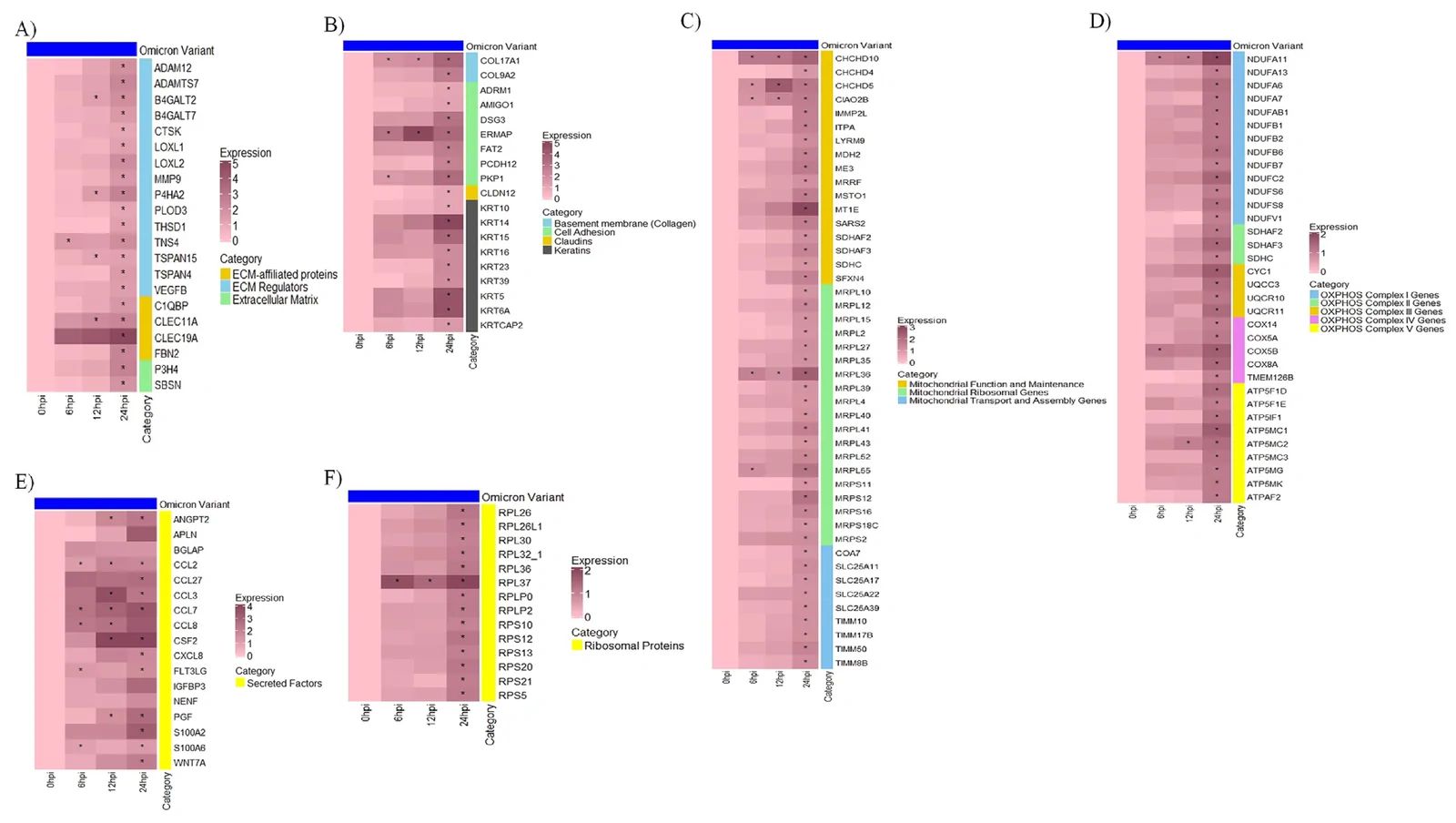
Heatmap showing the expression dynamics of genes associated with (**A**) extracellular matrix (ECM) organization and regulation, (**B**) basement membrane and cell adhesion, (**C**) mitochondrial function, maintenance, transport and assembly, (**D**) oxidative phosphorylation (OXPHOS) complex genes, (**E**) growth factors and secreted factors, and (**F**) ribosomal proteins following Omicron variant infection at 0, 6, 12, and 24 hpi. These genes represent upregulated DEGs identified in Profile 9. Genes with statistically significant expression changes (*p*-value ≤ 0.01) are indicated with asterisks (*).

In contrast, Profile 9 showed marked upregulation of genes involved in ECM organization, basement membrane integrity, cell adhesion, keratin proteins, growth factors, immune response, mitochondrial function, oxidative phosphorylation (OXPHOS), and ribosomal activity. These changes suggest active tissue repair, enhanced immune activation, and increased metabolic demand in response to infection. Basement membrane and cell adhesion genes (e.g., *COL17A1*, *COL9A2*, *DSG3*, *ERMAP*) were significantly upregulated in both variants, indicating efforts to maintain tissue integrity. Similarly, keratin genes (*KRT14*, *KRT5*, *KRT6A*) showed strong upregulation, reflecting epithelial repair and remodeling [37]. ECM remodeling was evident in both variants, with increased expression of regulatory genes such as *ADAM12*, *LOXL2*, and *B4GALT* family members, along with matrix metalloproteinase *MMP3*, suggesting active extracellular restructuring. ECM-affiliated proteins (e.g., *FBN2*, *MUC5B*) and related genes (*P3H4*, *SBSN*) were also consistently upregulated. Growth factors and secreted molecules were strongly induced, including *FGF23*, *TGFB2*, *GDF6*, *ANGPT2*, *APLN*, and *CSF2*, along with chemokines such as *CCL2*, *CCL7*, and *CXCL8*. These factors highlight enhanced inflammatory signaling, immune cell recruitment, and tissue repair processes in both variants, with minor differences in expression magnitude and timing. Notably, Omicron infection induced a stronger upregulation of mitochondrial, OXPHOS, and ribosomal genes compared to Delta. Genes involved in mitochondrial function (e.g., *IMMP2L*, *SFXN4*), electron transport chain complexes (e.g., *NDUF*, *COX*, *UQCR* families), and ribosomal proteins (*MRPL*, *MRPS*, *RPL*, *RPS*) were significantly elevated, indicating increased ATP production and protein synthesis [38]. In contrast, Delta showed relatively modest activation of these pathways. In the context of SARS-CoV-2 infection, marked differences were observed in the regulation of mitochondrial, oxidative phosphorylation (OXPHOS), and ribosomal genes between Delta and Omicron variants at 24 hpi. The Delta variant showed limited upregulation of mitochondrial genes, including *IMMP2L*, *SFXN4*, and *SLC25A17*, indicating moderate activation of mitochondrial function and cellular stress adaptation. In contrast, the Omicron variant induced a robust upregulation of genes associated with mitochondrial organization (e.g., *CHCHD*, *TIMM*, *SLC25* families), electron transport chain complexes (*NDUF*-Complex I, *UQCR*-Complex III, *COX*-Complex IV, and *ATP5*-Complex V), and metabolic regulation. This suggests enhanced oxidative phosphorylation and increased ATP production to meet elevated cellular energy demands [39]. Additionally, a pronounced upregulation of mitochondrial ribosomal (*MRPL*, *MRPS*) and cytosolic ribosomal (*RPL*, *RPS*) genes, along with translation initiation factors (*EIF* family), was observed in Omicron infection, indicating increased ribosome biogenesis and protein synthesis capacity. This likely supports both viral replication and host immune responses. Overall, these findings demonstrate that Omicron imposes a greater metabolic and translational burden on host cells compared to Delta, reflected by heightened mitochondrial activity and ribosomal function, representing an adaptive response to sustain energy production and protein synthesis during infection. Figure 7 and Figure 8 represent heatmaps of upregulated differentially expressed genes (DEGs) from Profile 9 across key functional categories in dog lung explant cultures infected with SARS-CoV-2 Delta and Omicron variants, including cell adhesion and structural components, extracellular matrix (ECM) organization and regulation, growth factors, secreted factors, mitochondrial function and transport, oxidative phosphorylation (OXPHOS) complexes, apoptosis, DNA repair, ribosomal proteins, and few others. The displayed genes correspond to upregulated DEGs from Profile 9. Statistically significant changes (*p* ≤ 0.01) are indicated by asterisks (*).

### 2.5. PPI Network Construction of Significant Profiles and Molecular Complex Detection (MCODE) Cluster Modules Identification

**Figure 9:**
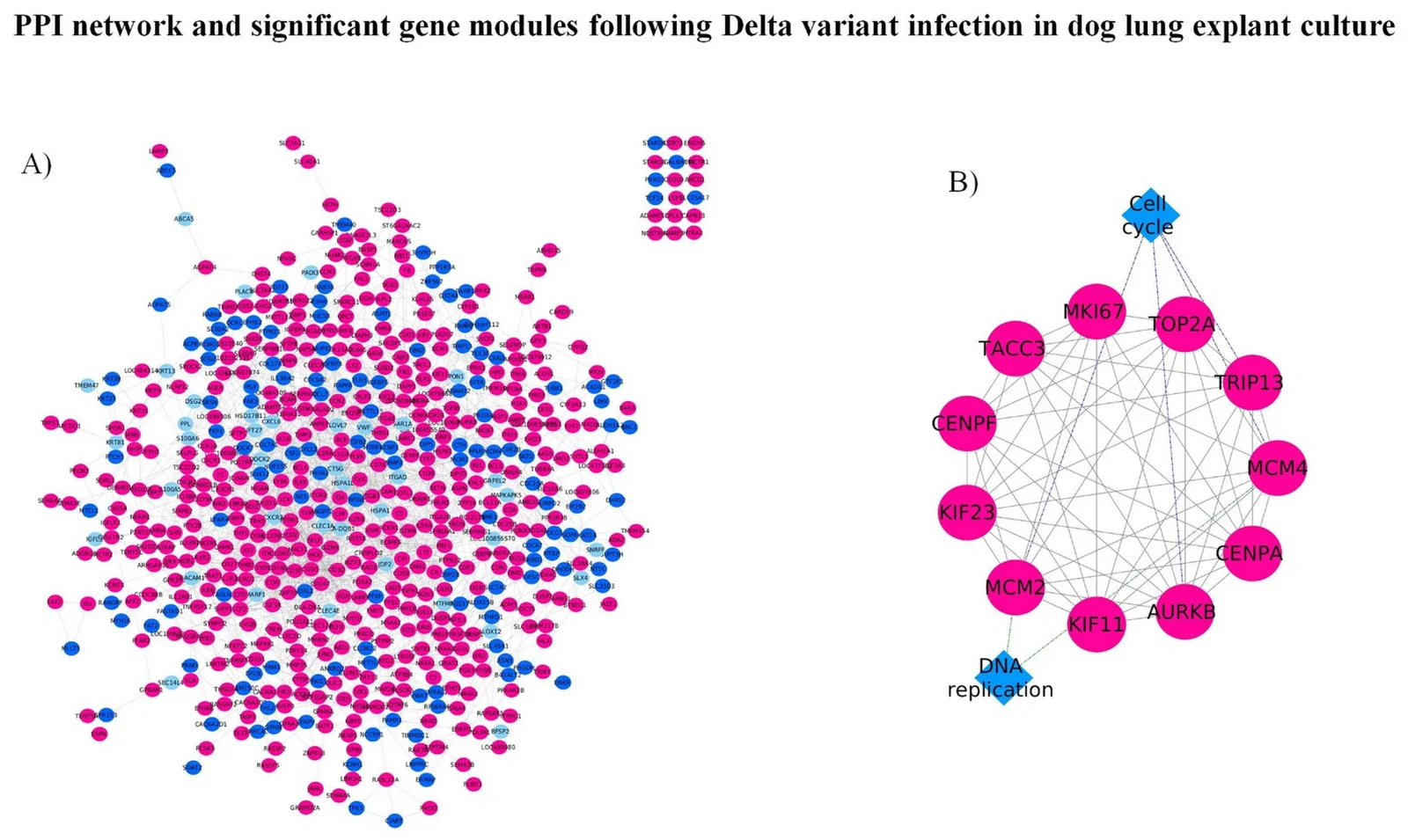
Protein–protein interaction (PPI) network construction and module analysis in dog lung explant cultures following Delta variant infection. (**A**) PPI network of genes from Profile 0 (pink) and Profile 9 (blue), visualized using Cytoscape. (**B**) Significant gene modules (MCODE score ≥ 10) identified from the PPI network, showing the associated gene groups.

**Figure 10:**
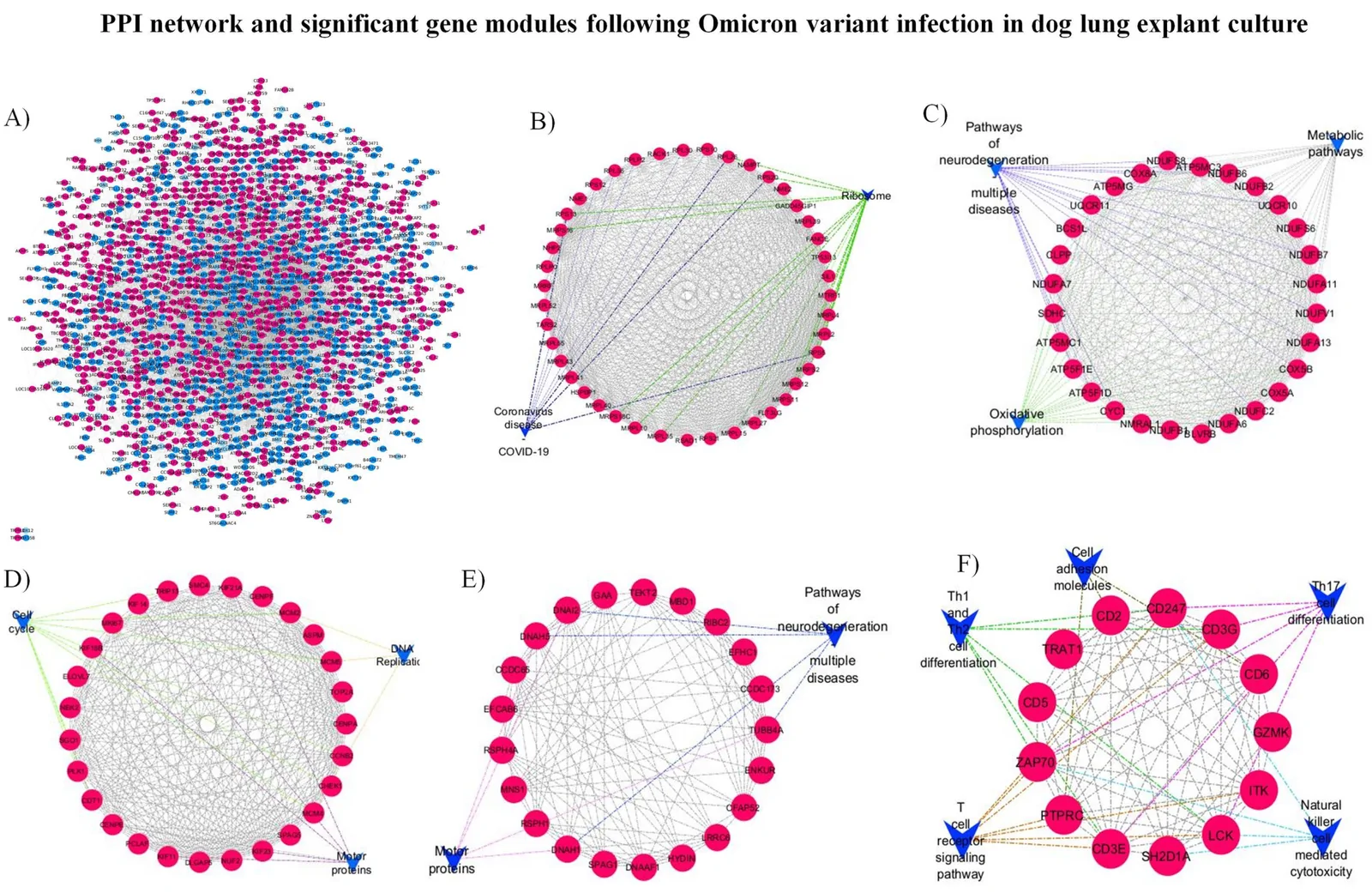
Protein–protein interaction (PPI) network construction and module analysis in dog lung explant cultures following Omicron variant infection. (**A**) PPI network of genes from Profile 0 (pink) and Profile 9 (blue), visualized using Cytoscape. (**B**–**F**) Significant gene modules (MCODE score ≥ 10) identified from the PPI network, showing the associated gene groups.

Protein–protein interaction (PPI) networks of genes belonging to the significant profiles were independently constructed for Delta (Figure 9A) and Omicron (Figure 10A) variant infections using STRING. The resulting networks were visualized in Cytoscape, and the most significant modules were identified using the MCODE plug-in. An MCODE score of ≥10 was used as the selection threshold. For the Delta variant, one significant module was identified (Figure 9B). Cluster 1 (MCODE score = 10; 11 nodes, 50 edges) was enriched in cell cycle and DNA replication pathways. In contrast, the Omicron variant exhibited five significant modules (Figure 10B–F). Cluster 1 (MCODE score = 33.42; 43 nodes, 702 edges) was enriched in ribosome and COVID-19-related pathways, while Cluster 2 (MCODE score = 22.37; 28 nodes, 302 edges) was associated with oxidative phosphorylation, pathways of neurodegeneration, and metabolic pathways. Cluster 3 (MCODE score = 20.46; 27 nodes, 266 edges) was linked to cell cycle and DNA replication, Cluster 4 (MCODE score = 10.90; 21 nodes, 109 edges) involved motor proteins and pathways of neurodegeneration, and Cluster 5 (MCODE score = 10.83; 13 nodes; 65 edges) was associated with the T cell receptor signaling pathway, Th1, Th2, and Th17 cell differentiation, natural killer cell-mediated cytotoxicity, and the cell adhesion molecules pathway.

### 2.6. Selection and Analysis of Hub Genes from Genes in Profile 0 and Profile 9

**Figure 11:**
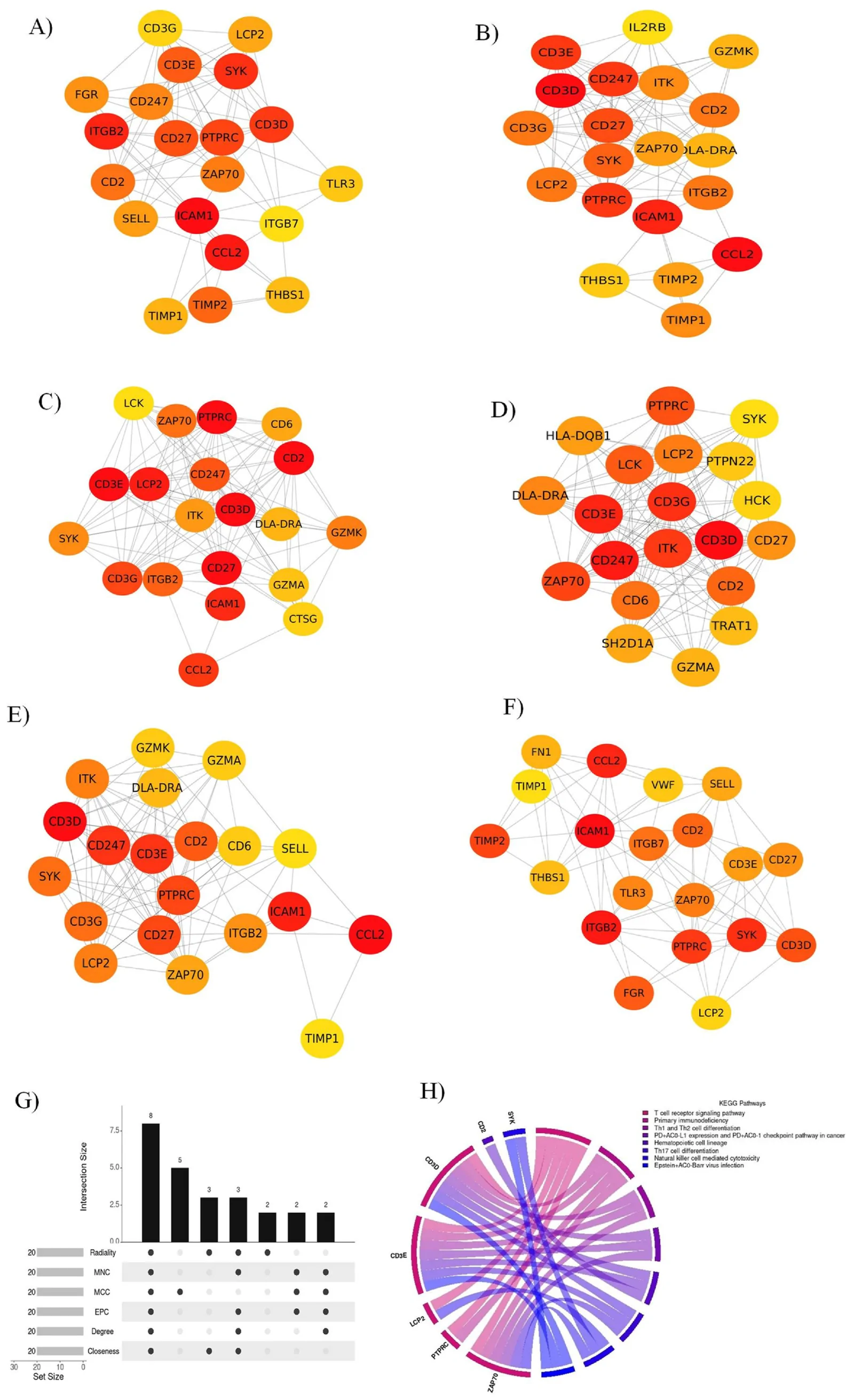
Identification of hub genes and their associated KEGG pathways in dog lung explant cultures following Delta variant infection using multiple algorithms. (**A**–**F**) Hub genes identified using six algorithms: MNC, MCC, EPC, Radiality, Closeness, and Degree. (**G**) UpSet plot illustrating the overlap among the six algorithms, identifying 8 common hub genes. (**H**) KEGG pathway enrichment analysis of the identified hub genes, showing their associated biological pathways.

**Figure 12:**
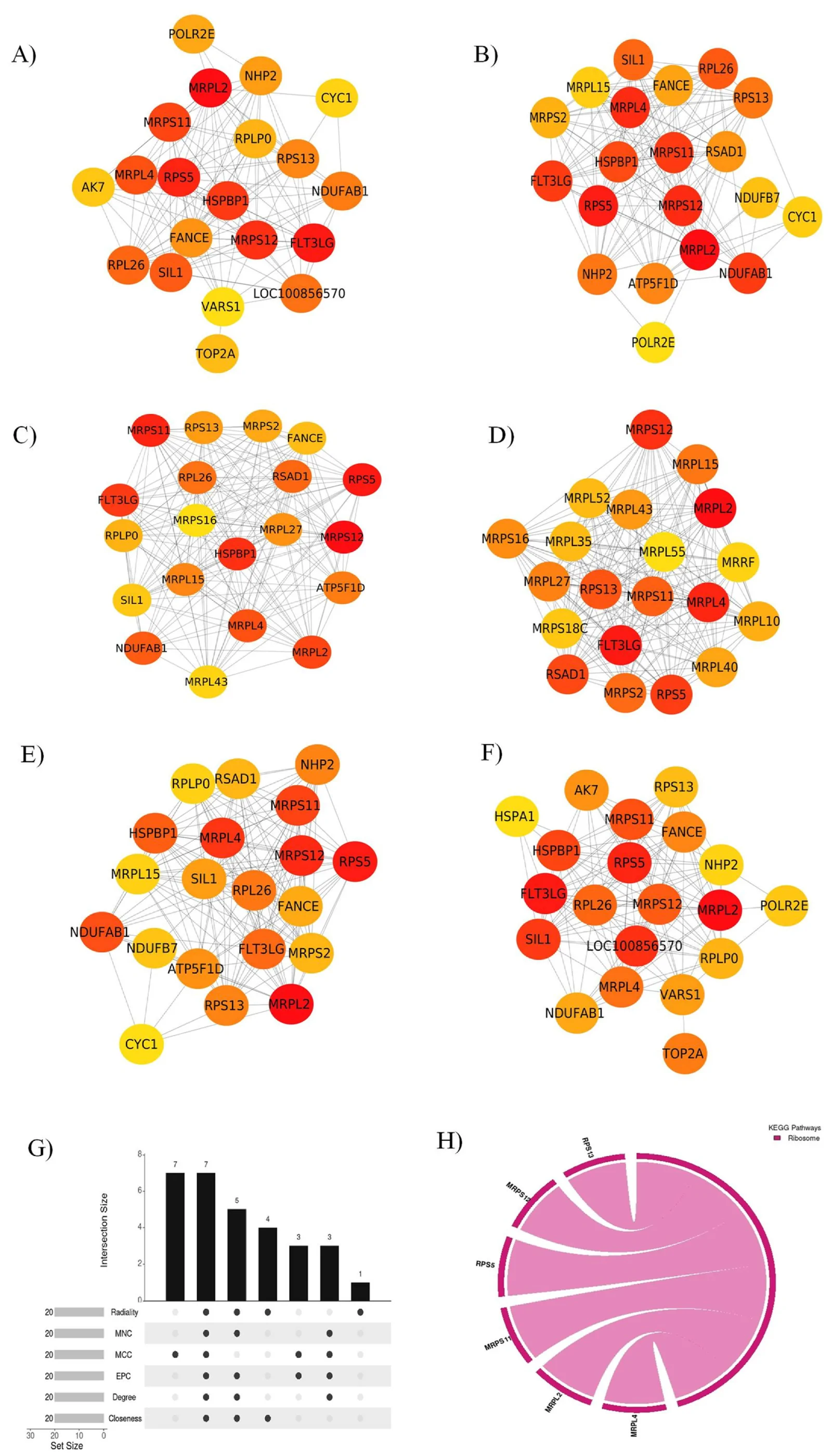
Identification of hub genes and their associated KEGG pathways in dog lung explant cultures following Omicron variant infection using multiple algorithms. (**A**–**F**) Hub genes identified using six algorithms: MNC, MCC, EPC, Radiality, Closeness, and Degree. (**G**) UpSet plot illustrating the overlap among the six algorithms, identifying 7 common hub genes. (**H**) KEGG pathway enrichment analysis of the identified hub genes, showing their associated biological pathways.

The top 20 hub genes were identified for the Delta and Omicron variants using six different algorithms (MNC, MCC, EPC, Radiality, Closeness, and Degree) implemented in the CytoHubba plugin (Figure 11A–F and Figure 12A–F, respectively). Each algorithm independently ranked hub genes based on distinct network topology parameters. To determine the genes consistently identified across all six algorithms, an UpSet plot was generated to visualize the intersections among the resulting gene sets. This approach enabled the identification of genes that exhibited consistent centrality across different ranking methods. For the Delta variant, eight hub genes were commonly identified by all six algorithms (Figure 11G), namely, *CD2*, *CD27*, *CD3D*, *CD3E*, *PTPRC*, *ZAP70*, *SYK*, and *LCP2*. Similarly, seven common hub genes were identified for Omicron variant infection (Figure 12G), including *FLT3LG*, *MRPL2*, *MRPL4*, *MRPS11*, *MRPS12*, *RPS13*, and *RPS5*. KEGG pathway enrichment analysis of the identified hub genes is presented in Figure 11H and Figure 12H for the Delta and Omicron variants, respectively. A comprehensive overview of all significantly enriched KEGG pathways (*p* ≤ 0.05) associated with hub genes, along with pathways corresponding to significant MCODE clusters (MCODE score > 10; *p* ≤ 0.05) for both variants, is provided in Supplementary Materials S2. The details of the hub genes identified following Delta and Omicron variant infections in dog lung explant cultures are summarized in Table 1.

### 2.7. Validation by qRT-PCR

**Figure 13:**
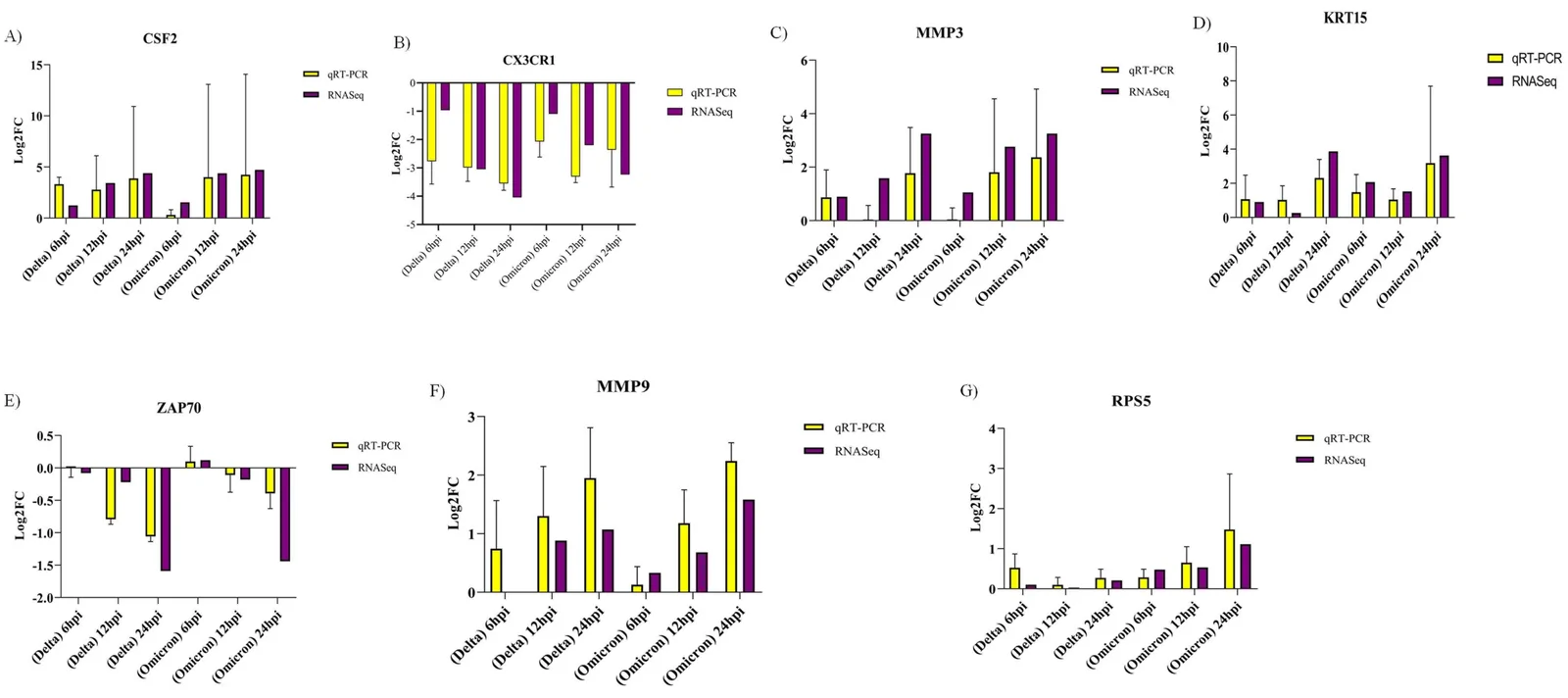
Validation of gene expression by qRT-PCR. Panels (**A**–**G**) display log_2_ fold-change (log_2_FC) values for genes validated in dog lung explant culture: (**A**) *CSF2*, (**B**) *CX3CR1*, (**C**) *MMP3*, (**D**) *KRT15*, (**E**) *ZAP70*, (**F**) *MMP9*, and (**G**) *RPS5*, with error bars representing standard error (SE).

For validation, seven genes were used, including *CSF2*, *KRT15*, *MMP3*, *MMP9*, *RPS5*, *ZAP70*, and *CX3CR1*. *GAPDH* and *ACTB* were used as housekeeping genes to normalize the qRT-PCR data. Real-time PCR results aligned with the RNA-Seq data. qRT-PCR bar plots with standard error is shown in Figure 13A–G.

## 3. Discussion

SARS-CoV-2 continues to infect a wide range of hosts, including humans, domestic animals, zoo animals, and wildlife, alongside the ongoing emergence of novel viral variants [4,36,47,48,49,50,51,52]. Domestic animals, owing to their close association with humans, may pose a potential risk for viral transmission and could contribute to the emergence of mutations and new variants. Reverse zoonotic transmission, in which humans transmit SARS-CoV-2 to animals such as dogs, has been extensively documented [53,54,55]. Experimental investigations have also demonstrated animal-to-animal transmission of SARS-CoV-2 in dogs [6,56], highlighting the importance of understanding the molecular mechanisms underlying viral infection in companion animals. Integrating transcriptomic analysis with ex vivo tissue models provides a valuable approach for investigating virus–host interactions, addressing the limitations of conventional in vitro systems and animal models, which often fail to adequately reproduce tissue-specific immune responses, particularly in the context of SARS-CoV-2 infection [57,58]. The pulmonary microenvironment is inherently heterogeneous, and variation in tissue structure and coexisting pathological features can influence the interpretation of molecular and tissue-level responses in lung tissue [59]. This complexity further supports the use of ex vivo models that preserve native tissue architecture and cellular interactions when investigating host responses to viral exposure. By maintaining tissue architecture and cellular complexity, these models facilitate a more physiologically relevant understanding of host immune responses and disease progression [32]. Building on our previous transcriptomic study in cats [31], the present study extends this investigation to dogs, aiming to characterize host gene expression changes following infection with SARS-CoV-2 Delta and Omicron variants. Notably, SARS-CoV-2 infection induces a strong inflammatory response that disrupts the extracellular matrix (ECM), a critical component for maintaining tissue integrity and facilitating repair [60,61]. In the respiratory system, the ECM provides structural support to the airways and forms the basement membrane in the lower respiratory tract and vasculature, playing an essential role in tissue homeostasis and development [62]. A prominent finding in both variants was the extensive downregulation of genes associated with extracellular matrix (ECM) organization and cell adhesion. Key structural components, including collagens (e.g., *COL6A5*, *COL11A2*), laminins, and fibronectin (*FN1*), were significantly suppressed, indicating disruption of lung tissue architecture. Additionally, integrins and ECM-regulatory proteins such as *ADAMTS1*, *ADAMTS4*, and *ADAMTS9* were downregulated, suggesting impaired ECM remodeling and turnover. These alterations are critical, as ECM integrity is essential for maintaining alveolar structure and facilitating immune cell interactions [63]. Similar ECM dysregulation has been reported in SARS-CoV-2 infection across species, including feline models [31], suggesting a conserved pathological response involving structural compromise of lung tissue. However, the observed expression changes do not independently establish structural damage, impaired matrix turnover, or functional disruption of the canine lung, as these outcomes were not directly assessed in the present study. At 24 h post-infection (hpi), the expression profiles also showed increased expression of several genes associated with ECM repair, epithelial integrity, and tissue remodeling. Basement membrane-associated genes (*COL17A1*, *COL9A2*) and cell adhesion molecules (*DSG3*, *ERMAP*) were significantly induced, indicating attempts to restore epithelial stability. The upregulation of claudins (e.g., *CLDN12*) further supports reinforcement of tight junctions, which are essential for maintaining barrier function and limiting viral spread [64]. Their upregulation may reflect changes in epithelial maintenance or remodeling pathways following viral exposure; however, it cannot be interpreted as direct evidence of restored barrier function or successful tissue repair without corresponding histological, protein-level, or functional assessments.

A striking feature of the reparative response was the robust induction of keratin genes, including *KRT5*, *KRT6A*, *KRT14*, and *KRT15*, particularly in Omicron infection. Keratins play a crucial role in epithelial regeneration and cytoskeletal stability [65]. *KRT14*, in particular, has been identified as a marker of lung regeneration during diffuse alveolar damage and acute respiratory distress syndrome [66]. The concurrent upregulation of *KRT15*, associated with epithelial–mesenchymal transition and tissue repair [37], suggests activation of regenerative pathways aimed at restoring lung integrity. Together, these findings suggest activation of epithelial repair and tissue remodeling pathways in canine lung explants following SARS-CoV-2 exposure. Whether these transcriptional responses translate into improved tissue recovery or reduced disease severity in vivo requires further investigation.

Another key distinction between variants was the differential regulation of immune responses. In Delta-infected samples, hub genes associated with T-cell receptor (TCR) signaling and adaptive immunity, including *CD2*, *CD3D*, *CD3E*, *ZAP70*, and *PTPRC*, were significantly downregulated. These genes are critical for T-cell activation and immune signaling, and their suppression suggests impaired adaptive immune responses. Such downregulation may represent a viral immune evasion strategy, consistent with reports that SARS-CoV-2 can inhibit T-cell activation pathways to facilitate persistence within the host [40,67]. Furthermore, reduced expression of signaling molecules such as *SYK* and *LCP2* may dampen inflammatory responses, potentially limiting excessive immune activation but also compromising viral clearance. *SYK* acts as an important mediator of the immune response in inflammatory and autoimmune diseases [68]. *SYK* inhibitor R406 reduces inflammatory cytokines and oxidative stress in DCs and neutrophils [69]. Similarly, *LCP2* plays an essential role in orchestrating effective antiviral immune responses, particularly by modulating immune cell activation and facilitating the recognition and elimination of infected cells [44].

In contrast, Omicron infection was characterized by a pronounced upregulation of genes involved in mitochondrial function, oxidative phosphorylation (OXPHOS), and ribosomal activity. Genes encoding components of the electron transport chain and mitochondrial ribosomes (*MRPL2*, *MRPL4*, *MRPS11*, *MRPS12*) were significantly elevated, indicating enhanced ATP production and metabolic activity. This is consistent with previous reports showing that SARS-CoV-2 exposure is associated with alterations in mitochondrial metabolism and cellular bioenergetics. In the present study, these transcriptional changes likely reflect host metabolic adaptation to viral-associated stress [39,70].

Additionally, the upregulation of cytosolic ribosomal proteins (*RPS13*, *RPS5*) suggests increased protein synthesis capacity, supporting host immune responses [38]. This metabolic reprogramming reflects a host adaptation aimed at sustaining cellular function under viral stress. Notably, such upregulation was more pronounced in Omicron compared to Delta, indicating a variant-specific shift toward metabolic dependence. The identification of *FLT3LG* as a hub gene in Omicron infection further highlights differences in immune regulation. *FLT3LG* plays a critical role in the expansion of dendritic cells and macrophages, supporting antiviral immunity. Studies in humanized models have shown that *FLT3LG* enhances myeloid cell-mediated interferon responses while maintaining immune balance through regulatory pathways such as the *USP18*–*ISG15* axis [45], suggesting modulation of innate immune pathways that may contribute to balanced immune responses following viral exposure. Importantly, the combined upregulation of mitochondrial and ribosomal pathways in Omicron-infected tissues indicates a coordinated response to sustain energy production and biosynthesis. These transcriptional changes may reflect cellular adaptation to viral-associated stress; however, their direct contribution to viral replication or tissue protection cannot be established from the present ex vivo model. In contrast, the immunosuppressive profile observed in Delta infection suggests a different pathogenic strategy focused on evading host immune responses.

Overall, the data reveal distinct transcriptional responses in dog lung explants following exposure to SARS-CoV-2 Delta and Omicron variants. Delta exposure was associated with downregulation of genes involved in adaptive immunity, particularly T-cell receptor signaling pathways, whereas Omicron exposure was characterized by increased expression of genes associated with mitochondrial function, ribosomal activity, and protein synthesis. These differences indicate variant-associated changes in immune-related and metabolic gene expression profiles. However, these transcriptional patterns cannot be directly interpreted as immune suppression, viral immune evasion, enhanced metabolic activity, or protective responses. Differences in viral burden, cellular composition, RNA quality, and stress associated with ex vivo tissue culture may also have contributed to the observed expression profiles. As disease severity, immune cell function, protein expression, and in vivo outcomes were not assessed, further studies are required to determine the functional and clinical significance of these findings.

Further insights into the host response were obtained through hub gene analysis. In dog lung explants infected with the Delta variant, key hub genes, including *CD2*, *CD27*, *CD3D*, *CD3E*, *PTPRC*, *ZAP70*, *SYK*, and *LCP2*, were found to be downregulated. This pattern indicates changes in the expression of genes associated with immune signaling and activation. These genes are integral to T-cell activation, signaling, and function, forming a critical component of the adaptive immune response. Their coordinated activity is essential for effective immune defense and proper intercellular communication within the immune system [43]. However, the observed downregulation does not establish reduced immune function, and the potential contribution of changes in immune cell composition requires further investigation. In contrast, Omicron variant infection in dog lung explants revealed a distinct hub gene profile, including *FLT3LG*, *MRPL2*, *MRPL4*, *MRPS11*, *MRPS12*, *RPS13*, and *RPS5*. Many of these genes are associated with mitochondrial ribosomal proteins and translational machinery. Previous studies have demonstrated that SARS-CoV-2 can disrupt mitochondrial function, impairing cellular respiration and host immune responses [46]. Additionally, *RPS13* and *RPS5*, components of the small ribosomal subunit, are essential for efficient mRNA translation, highlighting their dual role in supporting both host protein synthesis and viral replication [38]. The observed upregulation of these genes in dogs infected with the Omicron variant likely reflects a compensatory or adaptive response to viral-induced mitochondrial dysfunction. This response may help preserve cellular energy production and maintain homeostasis under conditions of viral stress, thereby enhancing cellular resilience. Overall, the findings of this study reveal distinct variant-specific host transcriptomic responses in canine lung explants following exposure to SARS-CoV-2. Delta exposure was associated with downregulation of genes involved in adaptive immune-related pathways, whereas Omicron exposure was characterized by enhanced expression of genes involved in mitochondrial function, oxidative phosphorylation, protein synthesis, and regulated immune-related processes. These differences indicate that the two variants elicit distinct patterns of host molecular responses. Furthermore, the coordinated expression of genes associated with epithelial integrity, tissue remodeling, and cellular metabolism suggests activation of adaptive cellular responses to viral-associated stress. The molecular pathways identified in this study provide a foundation for future investigations aimed at understanding the mechanisms underlying host responses to SARS-CoV-2 variants.

A limitation of the present study is that the mock-infected control collected at 6 h post-infection (hpi) was used as the baseline for comparisons with infected samples at 6, 12, and 24 hpi. Consequently, transcriptional changes observed at 12 and 24 hpi may reflect both virus-associated responses and time-dependent changes occurring during ex vivo tissue maintenance, including tissue degradation and culture-associated stress. In the absence of time-matched mock controls at each sampling point, these effects cannot be clearly distinguished, and the observed changes at later time points should therefore be interpreted cautiously. Future studies incorporating time-matched mock controls at 6, 12, and 24 hpi would help differentiate virus-associated transcriptional responses from temporal effects of ex vivo culture. Additionally, because disease severity, immune-cell function, protein expression, tissue pathology, and in vivo outcomes were not assessed, the functional and clinical implications of the observed transcriptional changes remain to be determined.

In conclusion, this study provides insights into variant specific transcriptomic responses of canine lung explants following exposure to the SARS-CoV-2 Delta and Omicron variants. Delta exposure was associated with greater downregulation of genes involved in immune-related pathways, whereas Omicron exposure was characterized by increased expression of genes associated with mitochondrial, oxidative phosphorylation, and translational pathways. Increased expression of genes associated with epithelial maintenance and tissue remodeling at 24 h post-exposure indicates transcriptional changes in pathways associated with these processes. Comparison with our previous transcriptomic study in cats [31] revealed differences in the transcriptional responses of companion animal species, highlighting the need for further comparative investigations. The identified hub genes and molecular pathways provide candidate targets for future mechanistic studies and independent validation. Because the present study was conducted using an ex vivo canine lung explant model and did not directly assess productive viral replication, the findings should be interpreted as early host tissue responses following SARS-CoV-2 exposure. Overall, this work expands our understanding of host virus interactions in dog lung tissue and provides a valuable resource for future studies investigating the molecular responses of companion animals to emerging SARS-CoV-2 variants.

## 4. Material and Methods

### 4.1. Source of Experimental Tissue

Lung tissue samples were collected from three recently deceased dogs (n = 3) that had died due to non-respiratory causes, including accidents, fractures, and other injuries, with no evidence of pre-existing pulmonary pathology. The samples were obtained in collaboration with AASRA, a non-governmental volunteer organization, and the State Veterinary Hospital, Bhopal, India. Following postmortem collection, the tissues were immediately placed on ice in RPMI medium supplemented with 2X concentration of antibiotics (gentamicin and antimycotic) and transported to the ICAR–National Institute of High Security Animal Diseases (NIHSAD) for further processing.

### 4.2. Explant Culture Preparation, Virus Source, and Infection

All experiments were conducted in a Biosafety Level 3 (BSL-3) containment facility at the National Institute of High Security Animal Diseases (NIHSAD), Bhopal, India. Lung tissues collected immediately after postmortem examination were washed twice with RPMI medium (Gibco, Thermo Fisher Scientific, Waltham, MA, USA; Cat. No. 11875093) supplemented with a 2X concentration of antibiotics, including gentamicin (Thermo Fisher Scientific, USA; Cat. No. 15710064) and antimycotic solution (Thermo Fisher Scientific, USA; Cat. No. 15240062). The tissues were then finely chopped into approximately 1 mm^3^ fragments using sterile curved surgical scissors in a Petri dish placed on ice to preserve cellular integrity. The resulting explant suspension was transferred into sterile tubes containing antibiotic-supplemented RPMI medium. A portion of the suspension was retained as a mock (non-infected) control, while the remaining explants were separately inoculated with SARS-CoV-2 Delta and Omicron variants at a multiplicity of infection (MOI) of 1. The viral stocks used for inoculation had been previously propagated in Vero E6 cells under biosafety level 3 (BSL-3) conditions. The explants were then seeded into 6-well culture plates at an approximate density of 10^7^ cells per well, based on the estimated cell yield from the processed tissue. The cultures were maintained at 37 °C in a humidified incubator with 5% CO_2_. Mock-infected control samples were harvested at 6 h post-infection (hpi) to establish the baseline for subsequent analyses. The 6 hpi mock control was used as the common baseline for all comparisons; therefore, time-dependent ex vivo culture effects at later time points were not independently evaluated. Infected explants were harvested at 0, 6, 12, and 24 h post-infection (hpi) and transferred into tubes containing TRIzol™ Reagent (Thermo Fisher Scientific, USA; Cat. No. 15596026).

### 4.3. RNA Isolation, Quantification, and qRT-PCR to Check Explant Culture

Total RNA was isolated from lung explant cultures collected at mock, 0, 6, 12, and 24 h post-infection (hpi) using the PureLink™ RNA Mini Kit (Thermo Fisher Scientific, USA; Cat. No. 12183018A) in accordance with the manufacturer’s instructions. RNA concentration was subsequently quantified using the Qubit™ RNA High Sensitivity (HS) Assay Kit (Thermo Fisher Scientific, USA; Cat. No. 2935440).

### 4.4. Checking Explants Using Real-Time PCR

qRT-PCR was conducted on the collected mock, 0 hpi, 6 hpi, 12 hpi, and 24 hpi samples using the VIRALDTECT-II Multiplex Real-Time PCR Kit for COVID-19 (Genes2Me, Gurugram, India; Cat. No. G2M020220), according to the manufacturer’s protocol. The kit contains specific primers and fluorescent probes targeting the *RdRp*, *E*, and *N* genes of SARS-CoV-2.

### 4.5. Data Generation

Approximately 500 ng of RNA isolated from mock, 6 hpi, 12 hpi, and 24 hpi samples was used for sequencing on the Illumina NovaSeq 6000 platform (Illumina, Inc., San Diego, CA, USA). Paired-end sequencing with a read length of 2 × 150 bp was performed, generating approximately 10 Gb of data per sample. Prior to sequencing, RNA quality and integrity were evaluated using NanoDrop spectrophotometry (Thermo Fisher Scientific, USA) by measuring the 260/280 and 260/230 absorbance ratios, along with 1% agarose gel electrophoresis. Three biological replicates were included for each experimental condition and time point. Detailed information on data generated is provided in Supplementary Materials S1.

### 4.6. Data Filtration and DEGs Identification

Raw sequencing reads from each sample were initially assessed for quality using FastQC v0.13.0 (Babraham Bioinformatics, Cambridge, UK). Adapter sequences and low-quality reads were subsequently trimmed using Cutadapt with default settings. The quality-filtered reads obtained from mock-infected and infected samples were then aligned to the Canis lupus familiaris reference genome. Gene-level read counts were estimated using HISAT2 and RSEM [71]. Differential gene expression analysis was performed using the DESeq2 package in R [72]. Genes meeting the criteria of a |log_2_ (fold change)| ≥ 1 for upregulated, ≤−1 for downregulated, and the *p*-value ≤ 0.01 were identified as DEGs. Raw *p*-values were used because application-adjusted *p*-values substantially reduced the number of DEGs, limiting downstream exploratory transcriptomic analyses. DEGs were identified and visualized using a volcano plot.

### 4.7. Short Time-Series Expression Miner Analysis

Short Time-series Expression Miner (STEM) software was employed to identify temporal patterns in gene expression during the course of infection by clustering genes based on the significance and similarity of their expression profiles [73]. The analysis was performed using 10 model profiles, with the maximum unit change between consecutive time points set to 1. Gene expression values were converted into log ratios relative to the control samples. Subsequently, genes were assigned to the predefined model profiles based on their expression patterns, and correlation coefficients were calculated to assess the similarity between observed and modeled profiles. Statistically significant profiles were highlighted in the resulting visualization, with profiles exhibiting similar expression patterns grouped for further analysis. Gene Ontology (GO) and Kyoto Encyclopedia of Genes and Genomes (KEGG) pathway enrichment analyses were conducted for the significant profiles using the DAVID database [74]. Enrichment terms with Benjamini-adjusted *p*-values and false discovery rate (FDR) values ≤ 0.05 were considered statistically significant.

### 4.8. Construction of a PPI Network, Module Analysis, and Hub Gene Identification

Protein–protein interaction (PPI) networks were generated using the Search Tool for the Retrieval of Interacting Genes (STRING) database for differentially expressed genes (DEGs) belonging to the significant STEM profiles (Profiles 0 and 9) identified in both Omicron and Delta variants. Interactions with a combined confidence score greater than 0.4 were considered for network construction. The resulting PPI networks were visualized using Cytoscape (v3.10.2). Significant network modules were identified using the MCODE plugin (v2.0.3) with an MCODE score threshold of ≥10 and the following parameters: degree cut-off of 2, maximum depth of 100, node score cut-off of 0.2, and k-core of 2. Hub genes were subsequently identified using the cytoHubba plugin (v0.1) based on their topological connectivity within the PPI networks. Six algorithms, namely Maximum Neighborhood Component (MNC), Maximal Clique Centrality (MCC), Degree, Closeness, Radiality, and Edge Percolated Component (EPC), were applied to identify candidate hub genes. The common hub genes identified across these algorithms were determined using an UpSet plot.

### 4.9. Validation Using RT-qPCR

To validate the RNA-seq findings, quantitative real-time PCR (qRT-PCR) was performed using the same RNA samples employed for library preparation. The assay was carried out using the NeoScript One Step qRT-PCR Kit with SYBR Green (Genes2Me; Cat. No. OS01) in accordance with the manufacturer’s instructions. Each 10 µL reaction mixture consisted of 5 µL of 2× One Step SYBR Green Mix, 0.5 µL of One Step SYBR Green Enzyme Mix, 0.5 µL each of forward and reverse primers (10 µM), and 3.5 µL of RNA template (15 ng). The thermal cycling protocol included reverse transcription at 50 °C for 3 min, followed by initial denaturation at 95 °C for 5 min and 30–35 cycles of denaturation at 95 °C for 10 s and annealing/extension at 60 °C for 30 s. Relative gene expression was quantified using the 2^−ΔΔCT^ method, with *GAPDH* and *ACTB* used as internal reference genes. Primers used in this study are listed in Table S3 of Supplementary Materials S1.

## Figures and Tables

**Table 1:** List of hub genes in SARS-CoV-2 Delta and Omicron variant infection in dog lung explants, including expression status and role.

Gene Symbol and Full Name	Status	Role	Hub Gene	
			Delta	Omicron
*CD2* (CD2 Molecule)	↓ (Down)	The *CD2* gene encodes a surface protein found on all peripheral blood T-cells. It plays a crucial role in T-cell adhesion and immune recognition by interacting with LFA-3 (CD58) and CD48, which helps facilitate the connection between T-cells and antigen-presenting cells (APCs) [40].	Yes	No
*CD3E* (CD3 Epsilon Subunit Of T-Cell Receptor Complex)	↓ (Down)	Plays an essential role in T-cell development. Defects in this gene cause immunodeficiency [40].	Yes	No
*CD3D* (CD3 Delta Subunit Of T-Cell Receptor Complex)	↓ (Down)	The *CD3D* gene is crucial for T-cell activation and signal transduction as part of the TCR/CD3 complex. It facilitates antigen recognition and immune response initiation, and is also essential for T-cell development in the thymus. Defects in *CD3D* can lead to immune deficiencies, affecting the body’s ability to fight infections [40].	Yes	No
*CD27* (CD27 Molecule)	↓ (Down)	*CD27* plays a critical role in regulating the immune response to COVID-19. Alterations in its expression levels may increase a patient’s susceptibility to severe disease complications [41].	Yes	No
*PTPRC* (Protein Tyrosine Phosphatase Receptor Type C)	↓ (Down)	The *PTPRC* (CD45) gene plays a crucial role in the regulation of immune responses. It is essential for T-cell and B-cell activation by facilitating antigen receptor signaling. CD45 also regulates cytokine signaling by inhibiting JAK kinases, helping to control immune cell activation, growth, and differentiation. Its function is critical for effective immune responses, as it ensures proper communication between immune cells during infection and inflammation [42].	Yes	No
*ZAP70* (Zeta Chain Of T Cell Receptor Associated Protein Kinase 70)	↓ (Down)	The *ZAP70* gene encodes a tyrosine kinase crucial for T-cell activation and signal transduction via the T-cell receptor (TCR). It plays an essential role in T-cell development, especially for CD8+ T-cells. Mutations in *ZAP70* can lead to severe combined immunodeficiency (SCID)**,** characterized by a lack of functional CD8+ T-cells and impaired immune response.	Yes	No
SYK (Spleen Associated Tyrosine Kinase)	↓ (Down)	Involved in coupling activated immunoreceptors to downstream signaling events that mediate diverse cellular responses, including proliferation, differentiation, and phagocytosis [43].	Yes	No
*LCP2* (Lymphocyte Cytosolic Protein 2)	↓ (Down)	*LCP2* plays a critical role in T-cell receptor signaling, NK cell activation, and the overall adaptive immune response. Proper function of *LCP2* is necessary for an effective antiviral response, influencing how well the immune system can recognize and clear infected cells [44].	Yes	No
*FLT3LG* (Fms Related Receptor Tyrosine Kinase 3 Ligand)	↑ (Up)	*FLT3LG* (Fms-like tyrosine kinase 3 ligand) is a cytokine primarily known for its role in hematopoiesis, where it supports the development, differentiation, and expansion of dendritic cells (DCs) and other immune cells [45].	No	Yes
*MRPL2* (Mitochondrial Ribosomal Protein L2)	↑ (Up)	*MRPL2* is important because mitochondrial dysfunction has been linked to COVID-19. Viruses like SARS-CoV-2 may target mitochondrial proteins, potentially leading to disrupted energy metabolism and impaired immune responses, making *MRPL2* crucial for maintaining mitochondrial function during infection [46].	No	Yes
*MRPL4* (Mitochondrial Ribosomal Protein L4)	↑ (Up)	The *MRPL4* gene encodes a protein that is part of the large subunit of the mitochondrial ribosome, essential for synthesizing proteins encoded by mitochondrial DNA. These proteins are crucial for mitochondrial function and energy production [46].	No	Yes
*MRPS11* (Mitochondrial Ribosomal Protein S11)	↑ (Up)	These genes encode a protein that is part of the small subunit of the mitochondrial ribosome, essential for synthesizing proteins within mitochondria. These proteins are crucial for mitochondrial energy production and cellular respiration [46].	No	Yes
*MRPS12* (Mitochondrial Ribosomal Protein S12)	↑ (Up)		No	Yes
*RPS13* (Ribosomal Protein S13)	↑ (Up)	The *RPS13* gene encodes a key protein in the small 40S ribosomal subunit, essential for protein synthesis by translating mRNA into proteins. It plays a structural role in ribosome assembly and function [38].	No	Yes
*RPS5* (Ribosomal Protein S5)	↑ (Up)	The *RPS5* gene encodes a protein that is part of the small 40S ribosomal subunit, crucial for protein synthesis. It helps assemble ribosomes and ensures proper mRNA translation. During SARS-CoV-2 infection, *RPS5* is used by the virus to produce viral proteins, essential for its replication. Disruption of *RPS5* may also affect the host’s ability to produce immune-related proteins, impacting the body’s defense against the virus [38].	No	Yes

## Data Availability

The datasets generated during the current study have been uploaded to the National Center for Biotechnology Information under Bio Project accession PRJNA1328388.

## Supplementary Materials

The following supporting information can be downloaded at: https://www.mdpi.com/article/10.3390/ijms27209319/s1.
