# Supplementary material for: Variant-Specific Temporal Transcriptomic Responses of Ex Vivo Dog Lung Explants to SARS-CoV-2 Delta and Omicron Infection
Source: Int J Mol Sci. 2026 Oct 20;27(20):9319. doi: 10.3390/ijms27209319 (PMC13705182; doi:10.3390/ijms27209319)
Supplement: Supplementary file 1 [file ijms-27-09319-s001.zip › Supplementary Materials S1.pdf]

Table S1: Ct values obtained for Delta and Omicron variant infected dog lung explant culture at different time points (control, 0hpi, 6hpi, 12hpi, and 24hpi) post infection.

Variant of SARS-CoV-2	Time point post infection	Dog		
		FAM (Target E gene)	Texas Red (Target RdRp gene)	Cy5 (Target N gene)
		Ct Values		
Delta Variant	Control_rep1	NA	NA	NA
	Control_rep2	NA	NA	NA
	Control_rep3	NA	NA	NA
	0hpi_rep1	23.01	21.64	21.80
	0hpi_rep2	22.89	21.53	21.58
	0hpi_rep3	22.91	21.74	21.92
	6hpi_rep1	19.05	19.19	19.05
	6hpi_rep2	19.09	18.15	18.15
	6hpi_rep3	19.32	18.26	18.76
	12hpi_rep1	20.15	19.06	18.89
	12hpi_rep2	19.21	18.02	18.05
	12hpi_rep3	19.96	19.52	19.10
	24hpi_rep1	19.67	18.59	19.05
	24hpi_rep2	19.58	18.33	19.07
	24hpi_rep3	20.10	19.15	19.35
Omicron Variant	Control_rep1	NA	NA	NA
	Control_rep2	NA	NA	NA
	Control_rep3	NA	NA	NA
	0hpi_rep1	24.43	22.62	21.87
	0hpi_rep2	24.21	22.61	22.07
	0hpi_rep3	30.90	30.37	29.52
	6hpi_rep1	22.63	20.90	20.76
	6hpi_rep2	22.93	20.13	19.29
	6hpi_rep3	22.41	19.83	19
	12hpi_rep1	22.26	20.26	18.85
	12hpi_rep2	22.14	20.33	19.54
	12hpi_rep3	21.83	20.17	19.39
	24hpi_rep1	22.06	19.58	18.45
	24hpi_rep2	21.90	19.69	18.61
	24hpi_rep3	21.91	20.16	19.11

Table S2: Transcriptomics raw data statistics.

SARS-CoV-2 Variant	Hours post infection (hpi)	Replicate No	Reads in millions	Raw data in GB
D E L T A	Control	1	6.2	10
		2	4.9	8
		3	7.4	12
	6hpi	1	5.4	8
		2	9.2	15
		3	7.7	12
	12hpi	1	5.2	8
		2	6.9	11
		3	6.4	10
	24hpi	1	10.5	17
		2	6.08	10
		3	7.8	12
O M I C R O N	6hpi	1	4.6	13.67
		2	3.2	9.58
		3	4.6	13.81
	12hpi	1	3.4	10.05
		2	4.3	12.80
		3	4.3	12.73
	24hpi	1	5.3	15.67
		2	3.9	11.93
		3	4.6	13.60

Table S3: List of primers used for qRT-PCR.

Sl. No	Primer Name	Sequence (5'-3')	Amplicon size	AT	References
1.	MMP9-F	CGCATGACATCTTCCAGTACCA	73bp	59°C	Saxena *et al.*, 2016
	MMP9-R	CCGAGAATTCACACGCCAGTA			
2.	RPS5-F	TCACTGGTGAGAACCCCCT	141bp	59°C	Tabtieang *et al.*, 2016
	RPS5-R	CCTGATTCACACGGCGTAG			
3.	MMP3-F	TGGACCTGGAAATGTTTTGGGT	125bp	59°C	Designed
	MMP3-R	GCAGCAACGAGGAATAAATTG5GTT			
4.	ZAP70-F	AAGCATAGCACGGAGAAGACA	240bp	59°C	Toyoda *et al.*,

	ZAP70-R	AGGTACTTCATCCCCATGGAC			2024
5.	KRT15-F	F GCCTCCTCTCTGGCAATGAG	85bp	59°C	Weiner *et al*., 2018
	KRT15-R	R TCCAGGGCGTGCACCTT			
6.	CSF2-F	TCTCTGAAGTGTTTGACCCTGA	102bp	59°C	Tamura *et al*., 2021
	CSF2-R	AAGGGATTCTTGAGGCTGGT			
7.	CX3CR1-F	TTTCTCCCGGACTGAATCAC	90bp	60°C	Maeda *et al*., 2012
	CX3CR1-R	GATGGACCCATCTCCATCAC			
8.	GAPDH-F	CTGGGGCTCACTTGAAAGG	72bp	59°C	Ayers *et al*., 2007
	GAPDH-R	CAAACATGGGGGCATCAG			
9.	ACTB-F	CAAATGTGGATCAGCAAGCAG	102bp	59°C	Nassiri *et al.,* 2018
	ACTB-R	GAAAGGGTGTAACGCAACTAAAG			

Table S4: AUC values for identified hub genes for (A) Delta and (B) Omicron variant infection in dog lung explant culture.

Gene symbol and full name	Hub gene	
	Delta (AUC)	Omicron (AUC)
CD2 (CD2 Molecule)	Yes (0.78)	No
CD3E (CD3 Epsilon Subunit Of T-Cell Receptor Complex)	Yes (0.70)	No
CD3D (CD3 Delta Subunit Of T-Cell Receptor Complex)	Yes (0.74)	No
CD27 (CD27 Molecule)	Yes (0.67)	No
PTPRC (Protein Tyrosine Phosphatase Receptor Type C)	Yes (0.81)	No
ZAP70 (Zeta Chain Of T Cell Receptor Associated Protein Kinase 70)	Yes (0.78)	No
SYK (Spleen Associated Tyrosine Kinase)	Yes (0.93)	No
LCP2 (Lymphocyte Cytosolic Protein 2)	Yes (0.89)	No
FLT3LG (Fms Related Receptor Tyrosine Kinase 3 Ligand)	No	Yes (1)
*MRPL2 (*Mitochondrial Ribosomal Protein L2)	No	Yes (0.81)
*MRPL4 (*Mitochondrial Ribosomal Protein L4)	No	Yes (1)
MRPS11 (Mitochondrial Ribosomal Protein S11)	No	Yes (0.67)
MRPS12 (Mitochondrial Ribosomal Protein S12)	No	Yes (0.96)
RPS13 (Ribosomal Protein S13)	No	Yes (1)
RPS5 (Ribosomal Protein S5)	No	Yes (1)

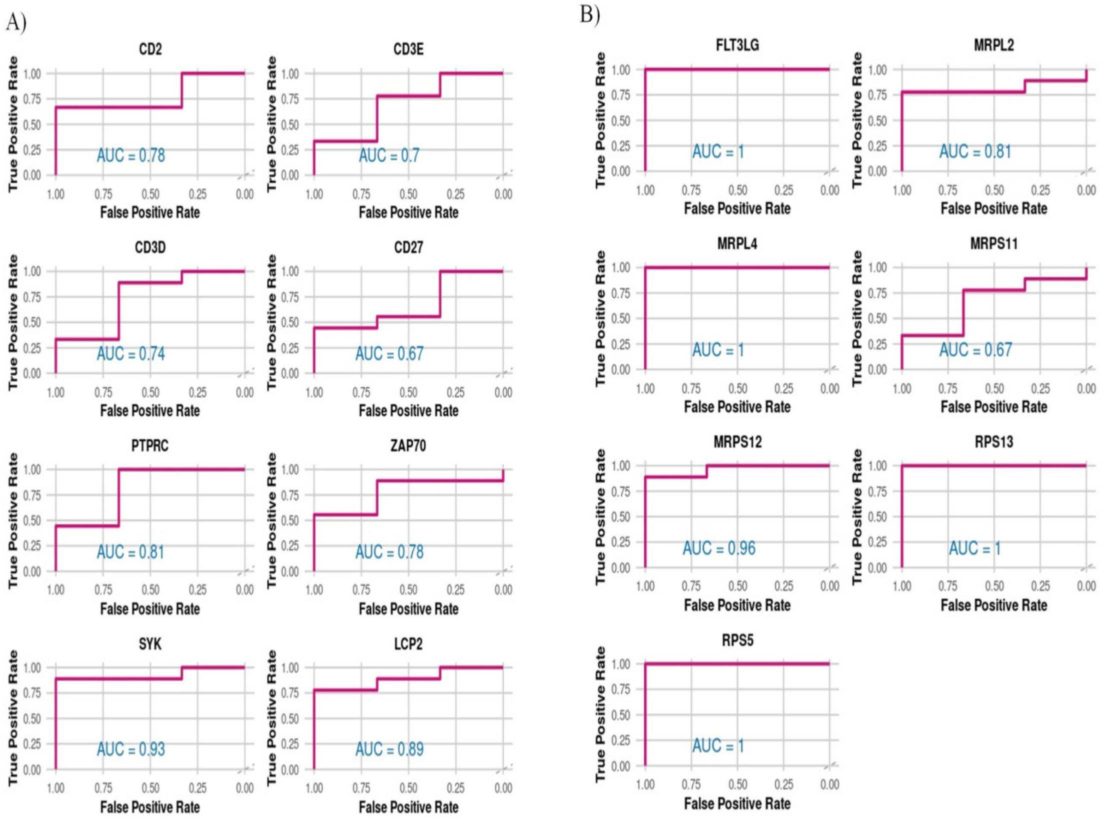

Supplementary Figure S1: AUC values for identified hub genes for (A) Delta and (B) Omicron variant infection in dog lung explant culture.
